# From explanation to intervention: Interactive knowledge extraction from Convolutional Neural Networks used in radiology

**DOI:** 10.1371/journal.pone.0293967

**Published:** 2024-04-10

**Authors:** Kwun Ho Ngan, Esma Mansouri-Benssassi, James Phelan, Joseph Townsend, Artur d’Avila Garcez

**Affiliations:** 1 Data Science Institute, City, University of London, London, United Kingdom; 2 Fujitsu Research of Europe Ltd, Slough, United Kingdom; Edith Cowan University, AUSTRALIA

## Abstract

Deep Learning models such as Convolutional Neural Networks (CNNs) are very effective at extracting complex image features from medical X-rays. However, the limited interpretability of CNNs has hampered their deployment in medical settings as they failed to gain trust among clinicians. In this work, we propose an interactive framework to allow clinicians to ask *what-if* questions and intervene in the decisions of a CNN, with the aim of increasing trust in the system. The framework translates a layer of a trained CNN into a measurable and compact set of symbolic rules. Expert interactions with visualizations of the rules promote the use of clinically-relevant CNN kernels and attach meaning to the rules. The definition and relevance of the kernels are supported by radiomics analyses and permutation evaluations, respectively. CNN kernels that do not have a clinically-meaningful interpretation are removed without affecting model performance. By allowing clinicians to evaluate the impact of adding or removing kernels from the rule set, our approach produces an interpretable refinement of the data-driven CNN in alignment with medical best practice.

## 1 Introduction

Convolutional Neural Networks (CNNs) excel at image recognition tasks. In recent years, these models prevailed in medical imaging research and applications. According to [1, 2], the performance of a CNN is now comparable to that of human radiologists. Historically, medical professionals discovered anomalies by analyzing visual patterns manually. As this is a time-consuming process, domain experts and researchers began investigating ways to automate the pattern identification process.

While CNNs continue to improve their state-of-the-art performance, the difficulty in interpreting the features extracted for prediction has limited their use in specialized applications such as medical diagnosis. Typically, model predictions are interpreted on an image-by-image basis by visualizing activated regions within a trained CNN, e.g. [3–5]. This type of interpretation can be highly subjective, and it is often insufficient to produce semantically rich reasoning from the visualization of activated regions. Additionally, semantically rich reasoning is required to understand the working processes of a trained CNN. This reasoning is typically symbolic, requiring the relationships between the extracted features (e.g. clinically-meaningful convolutional kernels) to be explicitly defined by symbolic rules representing the abstract concepts embedded in the features in compliance with domain best practices. Earlier work defined these concepts using pre-engineered features (such as radiomics [6, 7]) reflecting user-specified regions of interest. One of the aims of the present work is to automate the process of concept assignment using a CNN trained for object detection.

Prior research [8, 9] has investigated the abstract concepts extracted from kernels within the feature extraction layers of a CNN. Although it suggested that a number of kernels were related to objects, parts, materials and colors, the remaining kernels were uninterpretable. We argue that reliance on these uninterpretable kernels is contrary to medical best practice. If critical decisions are based on uninterpretable kernels, trust in the CNN predictions erodes.

The extraction of relevant knowledge from a trained CNN in the form of rules for reasoning also poses additional challenges. Deriving very large rule sets from CNNs limits model comprehensibility [10]. Similarly, methods for explaining individual images relying solely on heat maps fail to produce conceptual relationships of the CNN model [11]. In this work, we build on the previous research to extract rules from trained CNNs and investigate the semantically-meaningful relationships among the concepts referred to by these rules. The ERIC framework (Extracting Relations Inferred from Convolutions) [12] is used as the rule extraction method, as described below.

ERIC constructs a compact set of rules from a CNN by extracting logic programming rules from the CNN’s feature extraction layers. The rules offer a post-hoc decompositional explanation for image classification models. ERIC has been shown empirically to generate rules that can approximate the original CNN, as measured by high fidelity scores, i.e. the accuracy of the rules relative to the CNN rather than the data labels [12]. Each rule takes the form *L*_1_ ∧ *L*_2_ ∧ … ∧ *L*_*n*_ → *A*, denoting a conjunction of literals *L*_*i*_, 1 ≤ *i* ≤ *n* (each of which is either a propositional atom or its negation) that imply an atom *A* (if *L*_*i*_ hold true then *A* holds true). Each literal is associated with a CNN kernel, which in turn can be related to a concept by up-sampling kernels to input images. However, in the original ERIC approach [12] which relied on the visual inspection of relevant images, the manual concept assignment was regarded as a difficult process. A clustering technique was proposed in [13] to automate the concept assignment of kernels (these are heat maps known as kernel fingerprints obtained by up-sampling from the kernels back to the images). While this method made progress in defining concepts based on the most frequently activated regions of the entire image data set, it did not guarantee that these kernel fingerprints would reveal domain-relevant regions unless a deliberate attempt was made by human experts to define the regions. In this paper, we automate the localization of relevant regions based on an improved understanding of the regions. This is intended to improve domain relevance of the kernel concept descriptions used by the explanatory rules. Consequently, the kernel norm plot introduced in [13] can be used to quantitatively describe the concept (e.g. by associating concepts with radiomics features as discussed in the representative examples of this work). This should increase trust in the system by bringing it in line with best practices in specialized domains, as will be investigated in the sections that follow.

A user interface was also developed to display the reasoning of the extracted concepts from a trained CNN generated by ERIC so that clinicians can review the results and, if necessary, intervene in the set of rules to construct an alternative model that only uses meaningful and relevant concepts. This process will also be shown to be capable of removing irrelevant kernels without compromising model performance.

In addition, this work extends the experiments that evaluated the effect of the newly-defined kernel concept groups on model accuracy [13]. We shall demonstrate that these kernel groups within a CNN are not randomly learned, but they provide conceptual information essential to the performance of the model. This should help machine learning (ML) practitioners in streamlining CNN models in order to reduce the amount of computational resources required for CNN training and updates.

In summary, the contributions of this paper are as follows:

1. an application of knowledge extraction to enhance the explainability of features learned by CNN models used in radiology;
2. a method to evaluate the relationships between extracted features and radiomics features to produce meaningful concept descriptions of symbols extracted from the CNN;
3. a neuro-symbolic framework to enable domain expert validation and intervention when a trained CNN achieves high accuracy but for reasons that are not clinically justified.

The remainder of the paper is organized as follows: Section 2 summarizes related work that have led to the development of the proposed neural-symbolic approach. Section 3 discusses the clinical expectations for the deployment of AI systems in medical applications. Required background about symbolic rule extraction from CNNs and the ERIC framework is provided in Section 4. An overview of the proposed interactive explanation framework is provided in Section 5. Section 6 presents several representative examples and experimental results from the use of the proposed framework in medical imaging. Section 7 confirms our hypothesis by analyzing the effect of kernel concept groups on CNN performance. The paper concludes with a discussion and recommendations for future work in Section 8.

## 2 Related work

Traditionally, quantitative analysis and predictive models on images relied on manually extracted image features (such as texture, shape, and pixel intensity). This was also applied to medical images as a field of research known as radiomics [14]. Radiomics was defined by [6] as a high-throughput feature extraction process on user-specified regions of radiographic images. This extraction process however has been laborious and susceptible to human error in identifying targeted areas and the choice of features to apply. In addition, there was no standardized algorithm definition for image processing, resulting in challenges to the reproducibility and comparability of results [15]. Open-source algorithms, e.g. PyRadiomics in Python [15] and Radiomics in Matlab [16], were developed with the aim to mitigate such challenges. These algorithms are capable of extracting a large panel of engineered features from medical images. Second-order features, such as Gray Level Co-occurrence Matrix (GLCM) features, can also be derived from pixel intensity to account for the probabilistic correlation between neighboring pixels.

With the development of Convolutional Neural Networks (CNN), visual features could be automatically extracted from the convolutional and pooling layers using a data-driven gradient-based parameter search [17]. This automated feature extraction enabled trained CNNs to perform effectively across a range of predictive tasks, even in the specialized medical domain [1, 2]. However, the relationships between these learned features were encoded within the model’s large number of parameters. Numerous techniques for interpreting the internal feature representations of CNNs have been proposed, either by visualizing the highest activation of the hidden units at a network layer [18, 19] or by upsampling these representations to the resolution of the input images to identify these salient features [3, 5, 20]. These feature visualizations enabled the interpretation of relevant pixels in CNN classification. However, the conceptual interpretations from such visualizations tend to be subjective and may vary between different but related images.

The research in [8, 9, 21] examined the interpretation of CNN’s hidden units at a global level. Highly activated regions in a network layer were associated with interpretable concepts through a data set compiled for relating images with different concepts at various levels of abstraction (the *Broden* dataset). This approach demonstrated that a level of feature disentanglement could be achieved w.r.t. objects, object parts, texture, and color, despite some remaining uninterpretable. The evaluation was however confined to the lexicon of the Broden dataset, rendering concepts beyond the lexicon uninterpretable. While this strengthened the case for *semantic meaning* in CNNs, manually compiling a dataset of concepts remained laborious. In a specialized field, such as radiology, it may not be possible to specify the relevant concepts a-priori. In addition, understanding concepts in isolation may not be sufficient to explain how they relate to one another and the target output. In [22], it is also suggested that the compositional nature of logical concepts could closely approximate the network neuron behavior. Additionally, the study discovered that human-interpretable abstractions from the kernels in CNN image classification were positively correlated with model performance. Through the composition of concepts, they were also able to identify sources of adversarial examples for wrong predictions. Our purpose in this work of having logical rules *L*_1_ ∧ *L*_2_ ∧ … ∧ *L*_*k*_ → *t*_*i*_ is to specify that the combination of concepts *L*_1_, *L*_2_,…, and *L*_*k*_ imply a specific outcome, *t*_*i*_, and to use this knowledge to assist users in deriving the appropriate explanations.

Representation learning is a sub-field of machine learning that focuses on techniques for transforming raw data (e.g. image pixels) into the appropriate representation required by a learning model [23]. This data transformation automates the process of encapsulating the input data and other contextual information into a tabular form [24]. This tabular form can be regarded as a knowledge base that retains the semantic information about the given data. In [25], three levels of cognitive representations were introduced to bridge the gap between stored numerical representations for deep learning and symbolic representations for logic. At the neural network level, representations were embedded within the activation patterns of a densely connected network. The symbolic level encoded knowledge from logical rules through symbols and their relationships. A third (spatial) level, referred to as a conceptual space, represented data in geometrical, topological, or ordinal dimensions. Concepts were learned at this level by comparing data similarities to other data points within the conceptual space [25]. This general idea will be applied here in the context of our proposed approach for assigning radiomics concepts from activated regions of a trained CNN and mapping them onto symbolic rules.

Efforts have also been made over the years to convert knowledge encoded in a neural network into interpretable rules, which can be summarized in three broad classes of *knowledge extraction* methods [26]: (1) *pedagogical* methods explain the output in terms of the input without evaluating the network’s internal mechanisms; (2) *decompositional* methods divide the network to extract knowledge from its internal mechanisms (e.g. groups of neurons and weights); and (3) *eclectic* methods are those that combine elements of (1) and (2). Given the natural decomposition of functions within CNNs into *g*(⋅) and *h*(⋅) as mentioned in our problem setting, this work applies a decompositional method that is suitable for CNNs to achieve efficiency in the rule extraction process.

As an example of a closely-related pedagogical approach, [27] sought to mimic the input-output function of a neural network by building a soft decision tree based on the hierarchical probability distribution of a class. The decision tree did not rely on the hierarchical features within the network. While [27] reported high accuracy, their evaluation used soft targets from network predictions as training patterns. As a result, obtaining meaningful fidelity measurements was not possible in the case of [27].

ERIC yields global explanations for one or more convolutional layers as a decompositional rule extraction method. A quantization process was used to binarize kernels into logical literals. These literals were then used to generate symbolic rules by means of a logic program that approximates the behavior of the convolutional layer(s) with respect to the CNN’s output. The approximation M* of the original CNN was reported to achieve high classification accuracy and fidelity in [12]. The results were also evaluated in terms of the sizes of the extracted rule sets, with smaller sets considered to be more human-comprehensible.

A global layer-wise extraction of rules from CNNs was investigated in [28]. Outputs from kernels were translated into literals for the extraction of *M-of-N* rules, where a rule was interpreted as being *true* if and only if any combination of *M* literals out of a set of *N* literals is *true*. Kernels were represented by the outputs of neurons with the highest information gain. Rule extraction was accomplished using a heuristic search that prioritized literals based on the weights associated with the respective neurons leading to the target output. Although theoretically sound, large networks could render this approach inefficient.

[29] described a post-hoc approach in which representations were partly disentangled from the trained CNN and rearranged into a hierarchical AND-OR graph. Interpretability was illustrated qualitatively and quantitatively, but the explanations were not converted into a separate, simpler classifier. As a result, no fidelity evaluation could be conducted. This work was expanded in [30] to include the extraction of decision trees, with kernels specifically trained using a loss function proposed in [31].

We regard the ability to measure fidelity and to apply the extraction to any CNN, irrespective of the training protocol, as key requirements of any knowledge extraction. For this reason, this work is built upon the ERIC framework, taking also into consideration ERIC’s efficiency at extracting global rules from CNNs.

## 3 Clinician’s expectation on AI system adoption

In general, a conventional CNN model is regarded as a ‘black-box’ model in which the underlying mechanism for model prediction is too complex to interpret. This makes it difficult for clinical users to evaluate and justify the option of implementing an AI system into their professional workflow. [32], for instance, published the findings of a survey study of 690 radiologists (including 276 radiologists with practical clinical experience using AI-based algorithms) regarding their views on adopting AI systems to enhance their workflow. For the radiologists who had prior experience adopting AI systems, their primary use case scenarios for AI systems included (1) diagnostic interpretation, (2) image post-processing and quantitative evaluation, and (3) workflow prioritization. Moreover, 72% of them did not perceive integrating the AI system into their IT system/workflow as technically difficult, despite the fact that they may not be directly involved in the integration process.

82% of radiologists (out of a total of 171 respondents) who used an AI system to assist diagnostic interpretation deemed the prediction results to be reliable. However, only 25% reported a reduction in their workload due to the system’s assistive nature. Additionally, 86% of radiologists with practical AI experience (out of 111 respondents) would consider AI systems to be valuable in reducing workload for clinical workflow prioritization.

According to the survey, the biggest barriers to adopting a certified AI system include skepticism regarding the AI system’s added value and advertised performance, as well as concerns about increased workload resulting from the A.I. system adoption. The motivation for the proposed interactive model explanation framework will aim to address the use cases of assisted diagnostic interpretation and post-hoc analysis, as described in Section 5. It will convert a complex CNN model into a simpler, explainable model to improve effective communication, allowing radiologists to take on a more active role in the development and implementation of AI in their workflow, as recommended in [33]. The ultimate goal will be to produce a trusted AI solution that can be used for triage in a medical setting to enable first-pass clinical workflow prioritization.

## 4 Preliminaries on ERIC symbolic rule extraction

Let **x** denote a set of input images and **t** denote a set of target outputs, each indexed by the subscript *i*, where 1 ≤ *i* ≤ *n*. A convolutional neural network, *M*, is trained on examples {*x*_*i*_, *t*_*i*_} and consists of two parts: *g*(⋅) mapping *x*_*i*_ to the output of a feature extraction layer, call it *g*(*x*_*i*_), and *h*(⋅) mapping *g*(*x*_*i*_) to the CNN’s output, *h*(*g*(*x*_*i*_)). Let $A_{i,k}^{l}$ denote a matrix of activation values *g*(*x*_*i*_) at the feature extraction layer *l*, where 1 ≤ *k* ≤ *k*_*l*_ denotes a *kernel* of the CNN, represented by a square matrix of vectorized real numbers. Let $b_{i,k}^{l}$ denote a set of truth-values (*true* or *false*) assigned to each kernel (see Eq 1) by a function *Q* (see Eq 2) mapping the activation matrix to {−1, 1}, where −1 denotes *false* and 1 denotes *true*. $b_{i,k}^{l}$ can be expressed symbolically as either a positive literal $L_{i,k}^{l}$ when $b_{i,k}^{l}=1$, or a negative literal $\neg L_{i,k}^{l}$ when $b_{i,k}^{l}=-1$. In Eq 2, $a_{i,k}^{l}$ is the result of calculating the L1-norm of the kernels (kernel norms) from $A_{i,k}^{l}$ (see Eq 3), and $\theta_{k}^{l}$ is an user-defined threshold value calculated for each kernel. In this work, the mean L1-norm value for the entire training set was used (see Eq 4).

$$\begin{array}{c} b_{i,k}^{l}=Q\left(A_{i,k}^{l},\theta_{k}^{l}\right) \end{array}$$ (1)

$$\begin{array}{c} Q\left(A_{i,k}^{l},\theta_{k}^{l}\right)=\{\begin{array}{c} 1,\;\text{if}\;a_{i,k}^{l}>\theta_{k}^{l}\\ -1,\;\text{otherwise} \end{array} \end{array}$$ (2)

$$\begin{array}{c} a_{i,k}^{l}=\|A_{i,k}^{l}\| \end{array}$$ (3)

$$\begin{array}{c} \theta_{k}^{l}=\sum_{i=1}^{n}(a_{i,k}^{l})/n \end{array}$$ (4)

In ERIC, a set of symbolic rules *R* is generated as an approximation M* of *M* using a decision tree-based rule extraction algorithm similar to the C4.5 algorithm [34] trained on instances $\left\{b_{i,k}^{l},h\left(g\left(x_{i}\right)\right)\right\}$. Each rule *R*_*r*_ takes the form of a conjunction of literals $L_{1}\wedge L_{2}\wedge \ldots \wedge L_{k_{l}}$, obtained from the feature extraction layer, which implies a CNN classification target output *t*_*i*_, that is, $L_{1}\wedge L_{2}\wedge \ldots \wedge L_{k_{l}}\to t_{i}$. A rule defines a path from the root node to a leaf node in the extracted decision tree. Tree pruning is applied to prevent overfitting. The Gini index is used to determine the branching of tree nodes. If a leaf node has multiple outcomes following pruning, the majority class is selected as the prediction. The accuracy of the CNN is measured in the usual way as the percentage of input images that are classified correctly w.r.t. *t*_*i*_. The accuracy of the extracted rules is determined by the percentage of input images classified correctly by the rules also w.r.t. *t*_*i*_, i.e. the number of times that *R*(M*, *x*_*i*_) = *t*_*i*_ divided by the number of examples, where *R* denotes the extracted set of rules. The *fidelity* of the rules to the network is defined as the percentage of rule-based classifications that match the CNN’s classification as measured by *R*(M*, *x*_*i*_) = *h*(*g*(*x*_*i*_)). As illustrated in subsequent sections, qualitative evaluations of the rules are also performed by up-sampling and inspecting of literals in the rules against the input images.

## 5 Interactive model explanation and intervention

**Fig 1:**
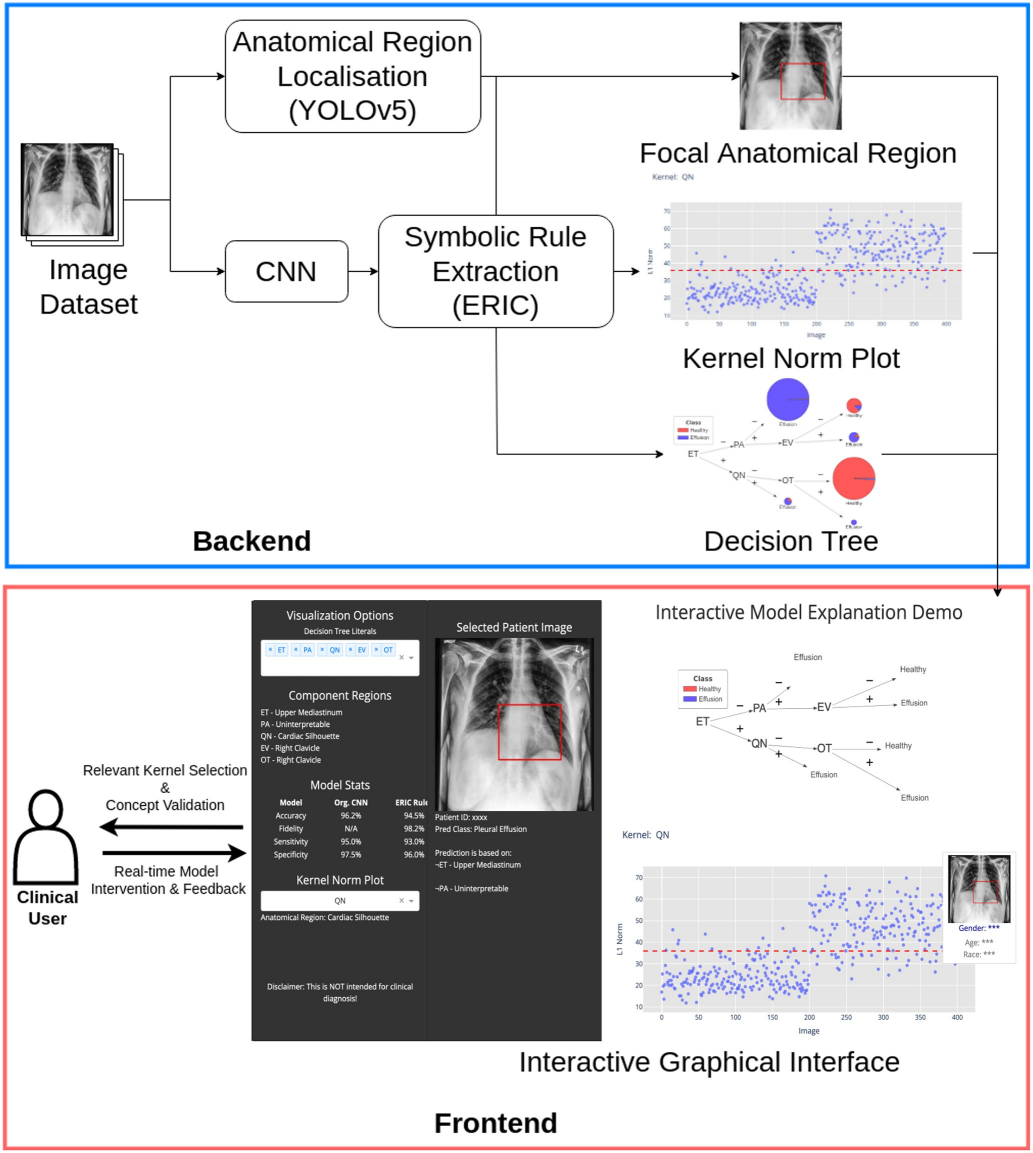
An overview of the interactive explanation framework, which includes (1) symbolic rule extraction, (2) anatomical region localization, and (3) graphical visualization of symbolic rule set for user interaction (see Fig 2 for higher quality diagram).

The proposed framework for interactive model explanation consists of (1) ERIC rule extraction, (2) automatic anatomical region localization for concept assignment, and (3) an interactive user interface. Fig 1 outlines the overview of the proposed interactive explanation framework. The data set used in this work is described in Section 5.1. The training of the CNN model for image classification is detailed in Section 5.2. Section 5.3 describes the mechanism of the ERIC framework for symbolic rule extraction from the trained CNN. Section 5.4 describes the parallel automated process for locating anatomical regions in frontal chest X-rays. This will be used to facilitate concept assignment by conducting a comprehensive analysis of the kernels used in the extracted rules. In Section 5.5, the features of the graphical user interface that promote human interaction and intervention will be discussed. Finally, Section 5.6 includes an evaluation of the relevance of these newly defined concepts to model performance.

### 5.1 Datasets

This work employed two data sets for (1) training a X-ray image classification model to detect pleural effusion from those that are healthy, and (2) training a model for anatomical region localization. All the X-ray images used in the training and subsequent analysis were in frontal view.

The CheXpert dataset [35] was used in task (1). Only frontal X-rays with labels as *pleural effusion* or *no finding* were selected. Images with the class *no finding* were deemed healthy. Images with artifacts or severely obstructed by supporting devices were removed. In addition, images should have a nearly square aspect ratio as input for the CNNs to minimize scaling distortion during training. 400 images were used for training, and 80 images were used for validation, with both classes being equally represented.

The NIH dataset [36] was used with reference to the study metadata in [37] for task (2). This dataset was used to train a CNN model (see Section 5.4) to locate nine anatomical regions within the frontal chest X-rays used in this work. These regions include (a) Trachea (T), (b) Upper Mediastinum (UM), (c) Cardiac Silhouette (CS), (d) Left Clavicle (LC), (e) Right Clavicle (RC), (f) Left Hilar (LH), (g) Right Hilar (RH), (h) Left Costophrenic Angle (LCA), and (i) Right Costophrenic Angle (RCA). The located anatomical regions were applied to the images of the CheXpert dataset used in the CNN classification model (see Section 5.2) to assign the associated anatomical regions as concepts (see Section 5.4).

### 5.2 CNN classification model training

A CNN classification model (*M*) based on the VGG-16 architecture was trained using the Adam optimizer and a learning rate of 10^−6^. The model was trained in batches of 32 images with the CheXpert dataset. Elite backpropagation (EBP) was also implemented to improve *class-wise activation sparsity* [38]. This was achieved by associating each class with a small number of kernels that were activated rarely but strongly for related images and assigning them as top kernels using a ranking and penalty function based on the activation probabilities of these kernels during training. EBP was previously demonstrated to result in a more distinct separation of kernel concepts and, arguably, more interpretable representations. When seeking to relate semantic meaning to kernels, the above separation of concepts through EBP can be very useful. The CNN model was trained using a random sample of input images for training (80%) and validation (20%), with each class equally represented.

### 5.3 Symbolic rule extraction

This work used the ERIC method [12, 39] for extracting rules from CNN kernels. Based on the ERIC framework, the last convolutional layer, *l*, of the trained VGG16, *M*, was quantized and binarized to produce literals. These literals were then used to derive rules as a measurable approximation M* of *M*. General details on the symbolic rule extraction is described in Section 4. It was empirically found that rules with a maximum of three literals in the body were sufficient to produce a good approximation, i.e. a high-fidelity score of the rules relative to the original CNN model. CNN kernels were assigned with semantic meanings through anatomical region localization and concept association as described in the next section (see Section 5.4). Fig 3a shows an example extracted rule, ¬ET ∧ ¬PA → Effusion, from CNN kernels ET and PA as a branch within a decision tree of rules.

### 5.4 Anatomical region localization and concept association

A segmentation model based on the YOLOv5x architecture [40] was trained independently using X-ray images from the NIH dataset [36] to locate 9 anatomical regions from individual frontal chest X-rays applied in the original trained CNN classification model. Anatomical regions were annotated with reference to [37]. The identified anatomical regions were superimposed on the activated image regions for each CNN kernel (i.e. 512 kernels at the last convolutional layer of a VGG16 model) to evaluate the region of interception. A hit was empirically considered if the interception over union (IOU) score was above 0.5 for each image. The region with the highest aggregated hit rate across the entire training dataset (and hits in at least more than 70% of the dataset) was regarded as the most frequently activated and representative anatomical region for the respective kernel. Additional criteria were implemented, including the restriction of anatomical regions highly hit by kernel activation to no more than two regions, to ensure the kernels were targeted to specific anatomical regions. This anatomical association offered a more comprehensible representation of clinical concepts than the kernel fingerprints used in previous work [13].

With the new representative anatomical regions serving as guiding points in the kernel norm plots, the concept expressed by the kernels could be interpreted with greater clarity. This process also allowed the filtering of uninterpretable kernels (i.e. those not associated with a specific anatomical region) for future research when more knowledge from medical image analysis is made known. To ensure the explainability of the extracted rules, only interpretable kernels were applied to generate the final clinically relevant rule set.

As only frontal X-ray images were used in this work, the relative positioning of the anatomical regions was consistent. This helped with the inspection and manual correction of any missing/incorrectly localized regions needed in this work. S1 Fig in S1 Appendix summarized the performance of the trained segmentation model in which the F1-score for all identified regions was high across a broad range of confident thresholds. The hilar regions and the costophrenic angles were among the more challenging regions, as the left and right regions were very similar. It should also be highlighted that the segmentation model was also capable of labeling the identified regions, albeit with weaker performance on the hilar and costophrenic regions as discussed earlier. S2 Fig in S1 Appendix displays sample X-ray images with annotated bounding boxes of the anatomical regions in healthy and pleural effusion cases of varying severity. Inferred outcomes for the CheXpert dataset were reviewed and manually corrected prior to further evaluation analysis such as the correlation with radiomics features.

### 5.5 Interactive user interface

**Fig 2:**
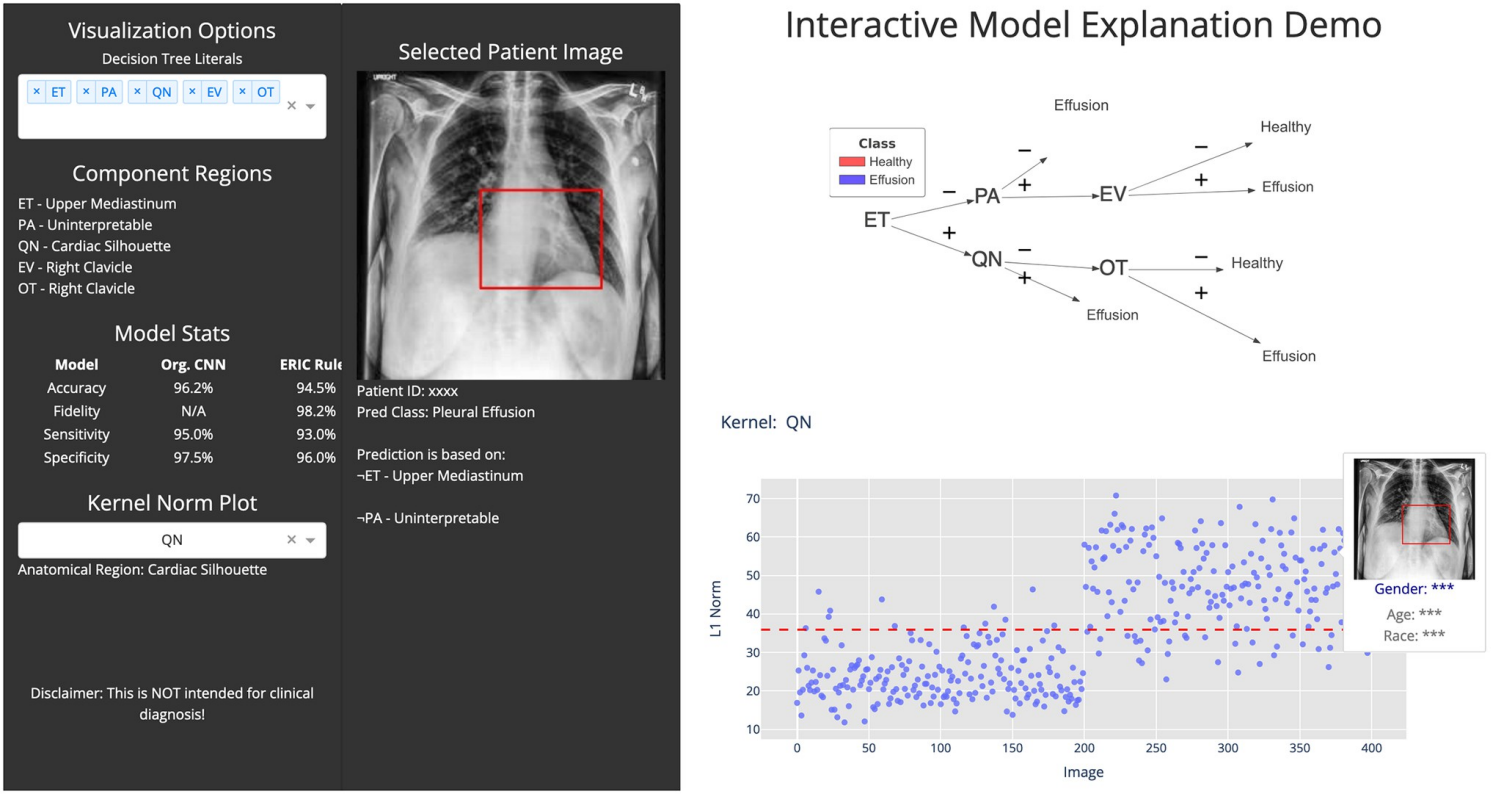
An interactive interface that facilitates clinical users to analyze and intervene with the model for improved clinical relevance. The grey sidebar (left) provides the user-defined kernel selection with the corresponding model output. The derived decision tree (see Fig 3a) displayed on the top right, and the kernel norm plot (see Fig 5a) for the selected kernel is at the bottom right for deeper analysis.

A graphical user interface was built to visualize the extracted symbolic rules as a decision tree (see Fig 2 top right). It also displays the represented anatomical region for each kernel in the decision tree. As illustrated in [13], the kernel norm plot (Fig 2 bottom right) was used to aid in assigning concept descriptions to kernels. A clinical user can hover over data points to obtain additional patient information and the corresponding X-ray image with the kernel representing the anatomical region overlaid (Fig 2 left). The interface allows the user to evaluate and modify the decision tree by selecting different kernels, enabling the user to interact with the system by asking questions such as “what happens if a kernel is replaced by another representing the same or another anatomical region?”.

### 5.6 Significance of anatomical kernel groups

This work extended the experiments in [13] where an analysis of concept clusters in the model’s performance was conducted. In previous work, an increasing group of kernels was randomly muted at 10% intervals (i.e. replacing the kernel outputs in the CNN with zero values). It was found that CNN model accuracy could be maintained even when up to 60–70% of the kernels were muted. In addition, activating/muting clusters of relevant kernels (i.e. kernels of specific kernel fingerprints) had a significant impact on the model’s accuracy against that of the original CNN despite the total number of relevant kernels used being less than the 40% of the randomly selected kernels needed to maintain model accuracy.

Three sets of investigations were conducted by activating or muting a combination of kernels relating to specific anatomical regions against other uninterpretable kernels according to the identified anatomical regions in Section 5.4 to determine the effect of anatomically relevant kernels on CNN model performance.

1. First, a comparison of the incremental activation of a combination of kernels from different anatomical regions was made in comparison with sets of randomly-selected uninterpretable kernels with equivalent set sizes over 100 sampling repetitions. This is to establish the significance of anatomically relevant kernels on model performance in comparison to random kernels.
2. Then, kernels corresponding to a specific anatomical region were muted, along with all uninterpretable kernels. This evaluated the dependence of other relevant kernels on those of the selected anatomical region in order to maintain the accuracy of the trained CNN.
3. Lastly, an evaluation of a combination of kernels from four anatomical regions was undertaken, as it was found that a decision tree with four anatomical regions was sufficiently accurate to represent the classification examples.

The next sections discuss the results of the above investigations.

## 6 Representative examples of interactive model explanation

As described in Section 5.1, a CNN classification model was trained to detect pleural effusion from frontal chest X-rays using the CheXpert dataset. Pleural effusion is defined as the abnormal accumulation of fluid in the pleural space, typically observed in the lower lung zones of an X-ray. It can be caused by various underlying diseases, such as pneumonia, congestive heart failure, and malignancy [41]. The chosen trained CNN model has a sensitivity of 95.0% and a specificity of 97.5%.

**Fig 3:**
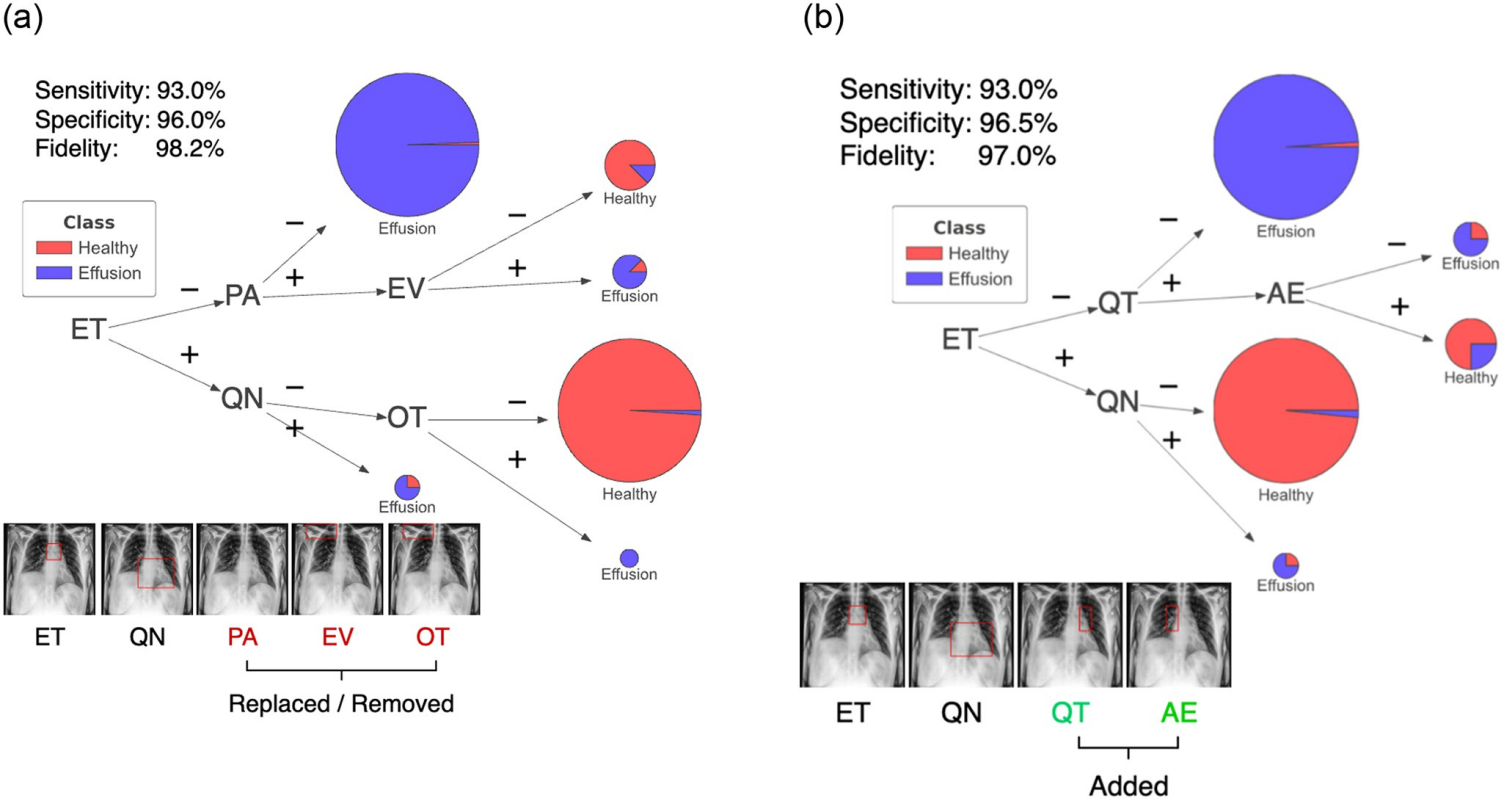
(a) Initially generated rule set from trained CNN in the form of a decision tree. (b) Human intervened clinically relevant rule set in the form of a decision tree. This example illustrates the effect of human intervention to improve the clinical relevance of constituent kernels for pleural effusion while maintaining model performance. The resulting kernels consist of anatomical regions—Upper Mediastinum (ET), Left Hilar (QT), Right Hilar (AE) and Cardiac Silhouette (QN). Kernels that were uninterpretable or irrelevant (such as PA, EV and OT) were discarded.

**Fig 4:**
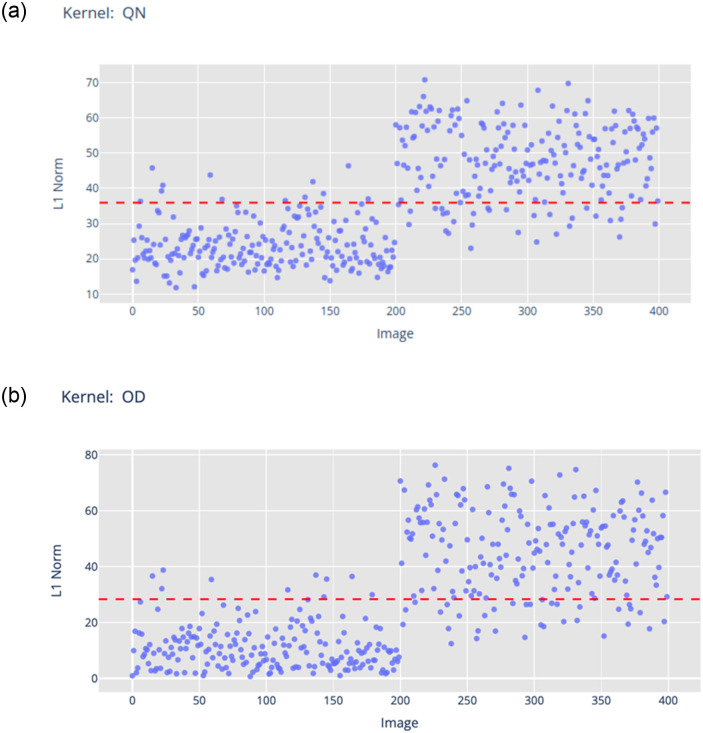
The kernel norm plot (L1-norm values) for (a) kernel QN and (b) Kernel OD, both of which represent the Cardiac Silhouette. The first 200 data points from the training dataset are labelled as *healthy* and the next 200 as *pleural effusion* according to the ground truth. A threshold value (red line) separates positive literals (e.g. QN) (above the line) and negative literals (¬QN).

**Fig 5:**
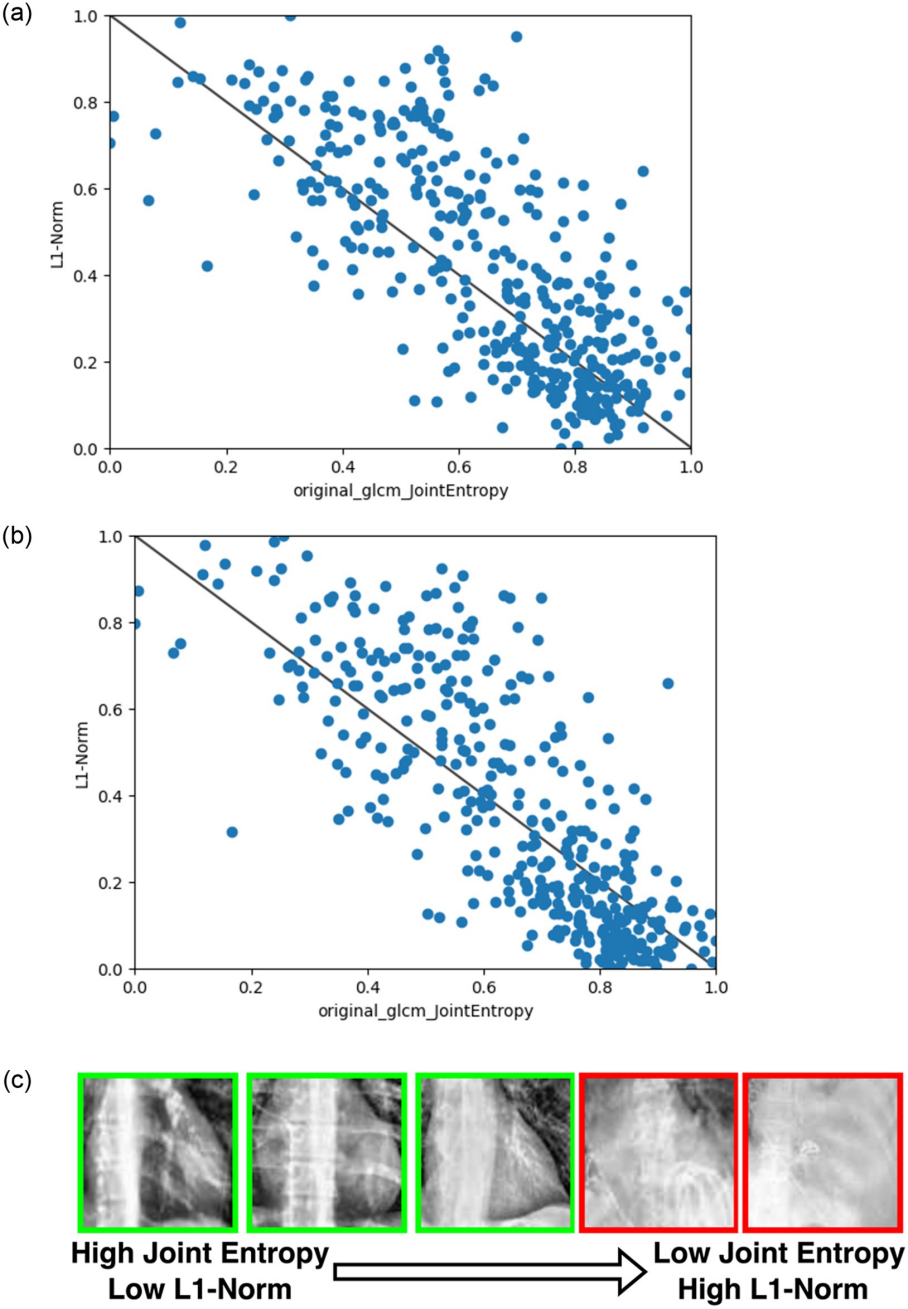
A negative correlation between Joint Entropy (GLCM) with L1-Norms for (a) Kernel QN and (b) Kernel OD. Sub-figure (c) shows images of the Cardiac Silhouette region sorted row-wise from highest joint entropy (left) to lowest joint entropy (right). Those images with *healthy* as ground truth label are outlined in green while those with *pleural effusion* are outlined in red.

An initial set of symbolic rules was extracted from the trained CNN using the ERIC framework (see Fig 3a). Five kernels were required (labeled here with associated anatomical regions): ET (Upper Mediastinum), QN (Cardiac Silhouette), PA (Uninterpretable), EV and OT (both on the Right Clavicle). This set of rules has a sensitivity of 93.0% and a specificity of 96.0%. It also has a fidelity of 98.2%. Nevertheless, this set of rules included an uninterpretable kernel (PA), and two irrelevant kernels (EV and OT—Right Clavicle—which is located at the upper lung zone in an X-ray). A further visual inspection of kernels via the kernel norm plot (see Figs 4a & 5a) revealed that kernel QN distinguishes pleural effusion cases from healthy cases based on features within the anatomical regions of the cardiac silhouette and the lung space borders. The presence of pleural effusion will obscure the lung space and the border of the left ventricle of the heart commonly understood as the “silhouette sign”. In contrast, no visible imaging pattern targeting pleural effusion was identified in kernels PA, EV and OT at the most frequently activated regions of each kernel. However, the region of the right clavicle bone from kernel EV or OT may be useful for determining the patient’s image-capturing position [42, 43].

Upon review by an experienced medical professional, kernels PA, EV and OT could not be assigned any meaningful connection to a diagnosis of pleural effusion and so were also regarded as irrelevant. However, new kernels (such as QT and AE relating to the left and right hilar) could be plausibly linked to a diagnosis of pleural effusion and therefore these were included.

The modified rule set had a sensitivity of 93.0% and a specificity of 96.5%. The fidelity of this rule set remained high at 97.0%. This modified rule set can be said to produce a more clinically relevant explanation, as it is supported by evidence from other independent medical research findings [41, 44, 45] and it maintains classification performance. Fig 3 presents the modification and the corresponding kernels used in the rule set.

**Fig 6:**
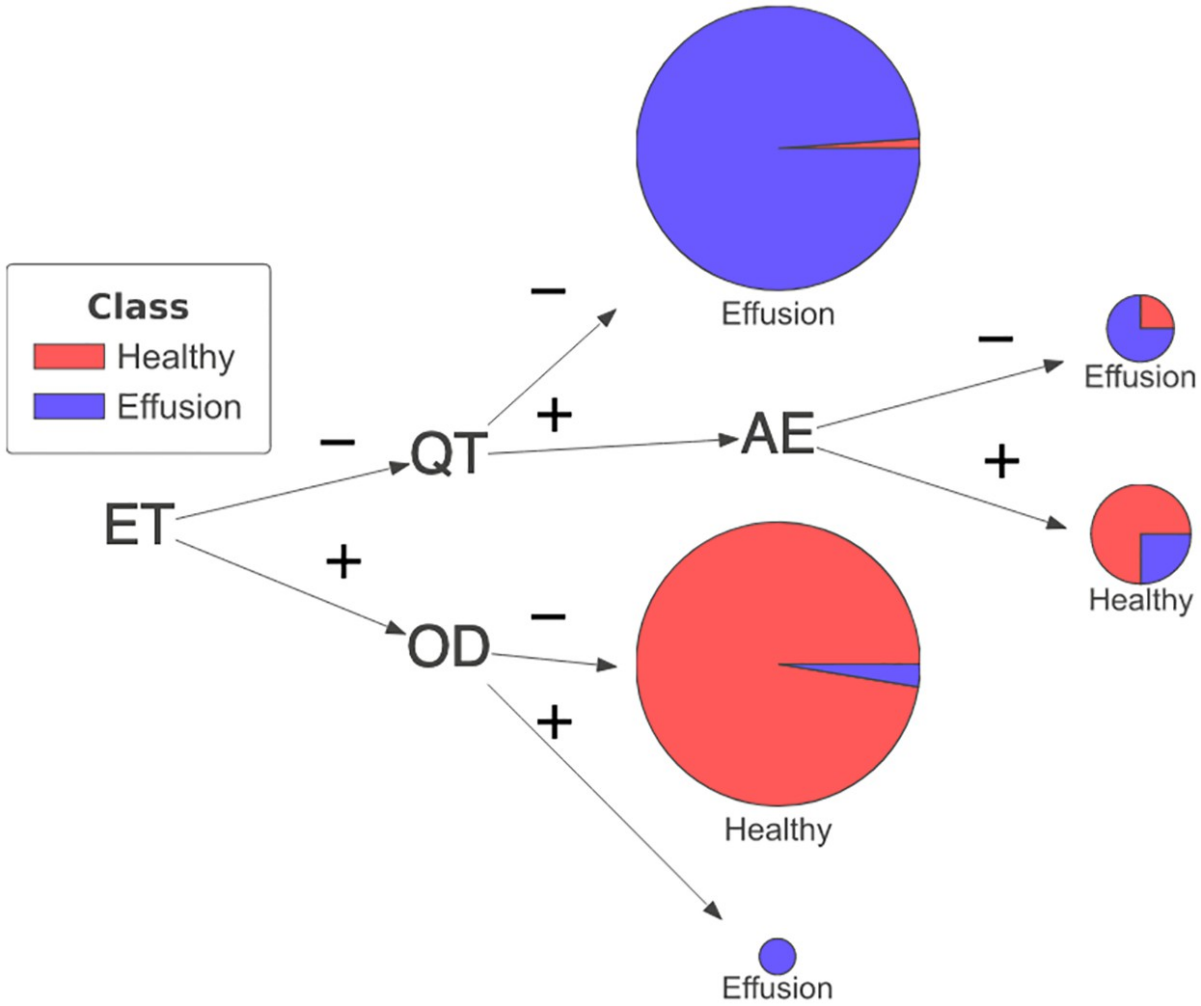
An example of rules generated by changing a single kernel for the Cardiac Silhouette region (i.e. from kernel QN to kernel OD).

An alternative rule set (see Fig 6) demonstrated that altering the kernel associated with the cardiac silhouette region from QN to OD did not compromise performance (sensitivity of 92.0% and specificity of 97.0%). Fidelity to the original CNN model remained consistent at 96.8%. Additional examples of the impact kernel changes for the hilar regions are provided in S2 Appendix, all of which consistently exhibit high accuracy.

On the selected kernels, an additional radiomics texture analysis was performed using the pyradiomics package [15] to provide a quantitative justification for the visual changes observed in the kernel norm plots. For instance, the L1-norm values for kernel QN and OD were highly correlated with the Joint Entropy in the Gray Level Cluster Matrix (GLCM) as shown in Fig 5a and 5b. Joint entropy could be viewed as a quantifiable measure of the randomness/variability of pixel intensity in relation to its spatial neighborhood. Low entropy values indicated a more homogeneous texture and vice-versa (see Fig 5c in comparison with S6(c) Fig showing the representative examples of the full spectrum). This validated the visual changes that a user will observe when hovering over the images in the kernel norm plot across the healthy and pleural effusion cases. Detailed analysis, including additional results for the Upper Mediastinum, both Left and Right Hilars, are presented in S2 Appendix with similar conclusions that the change in kernel norm values could be reflected by the change in image texture (radiomics features) at the specified anatomical regions.

**Fig 7:**
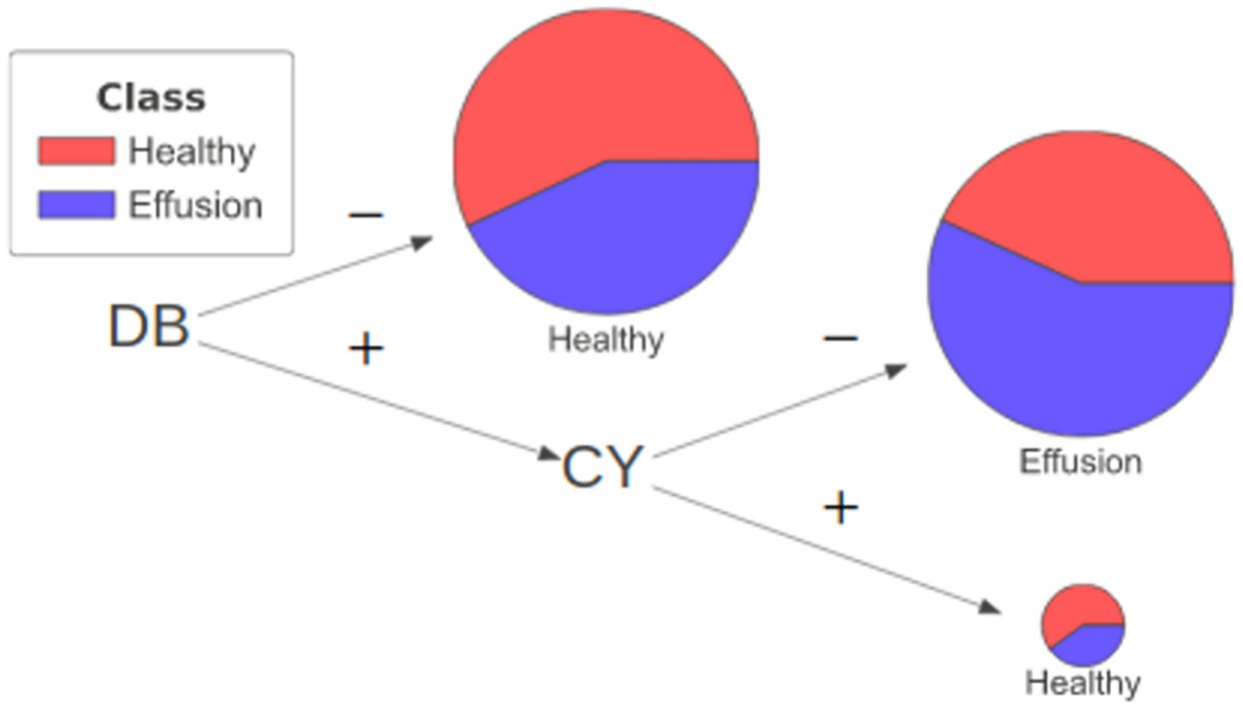
An example of rules generated by kernels that are not targeting any anatomical region (i.e. less than 5 images are activated that match any anatomical regions).

Lastly, a set of rules constructed by uninterpretable kernels (see Fig 7) with no more than five images activated for any anatomical region (i.e. <1.25% of image data) was used to illustrate the opposite extreme of applying uninterpretable/irrelevant kernels to form an explainable rule set. Such a rule set yielded a sensitivity of 48.5% and a specificity of 64.0%. It could be hypothesized that these kernels did not acquire any task-relevant knowledge during CNN model training. These kernels might be disregarded. Similar conclusions were observed in [13], where a CNN model using only redundant kernels was found to produce inaccurate predictions.

**Fig 8:**
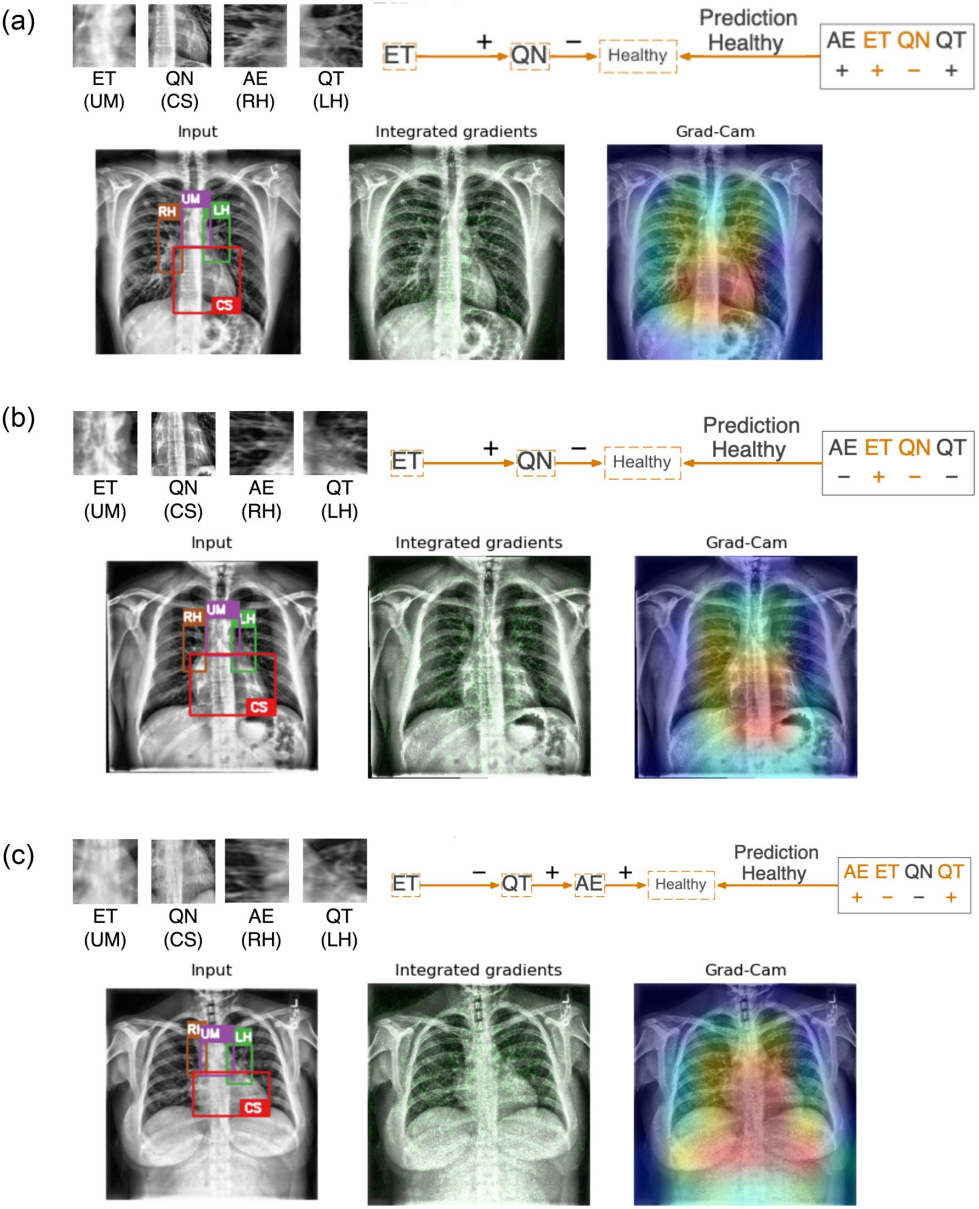
Three representative examples of applying neural-symbolic rules on healthy images in the training images. (a) The prediction is attributed to a clear Upper Mediastinum (in purple box with literal *ET*) and the absence of obscurity at the Cardiac Silhouette (in red box with literal ¬*QN*). (b) The prediction in Example #2 also relies on similar observations at the Upper Mediastinum (literal *ET* and Cardiac Silhouette (literal ¬*QN*). (c) Example #3 illustrates a healthy case where the Upper Mediastinum has a more homogeneous texture (¬*ET*) and the prediction utilized the clear visibility of structure at the Left and Right Hilar (in green box with literal *QT* and in brown box with literal *AE*). Integrated gradient and Grad-CAM highlight the significance of the mediastinum (in green and red respectively) without further details and reasoning on the predictions as being healthy.

**Fig 9:**
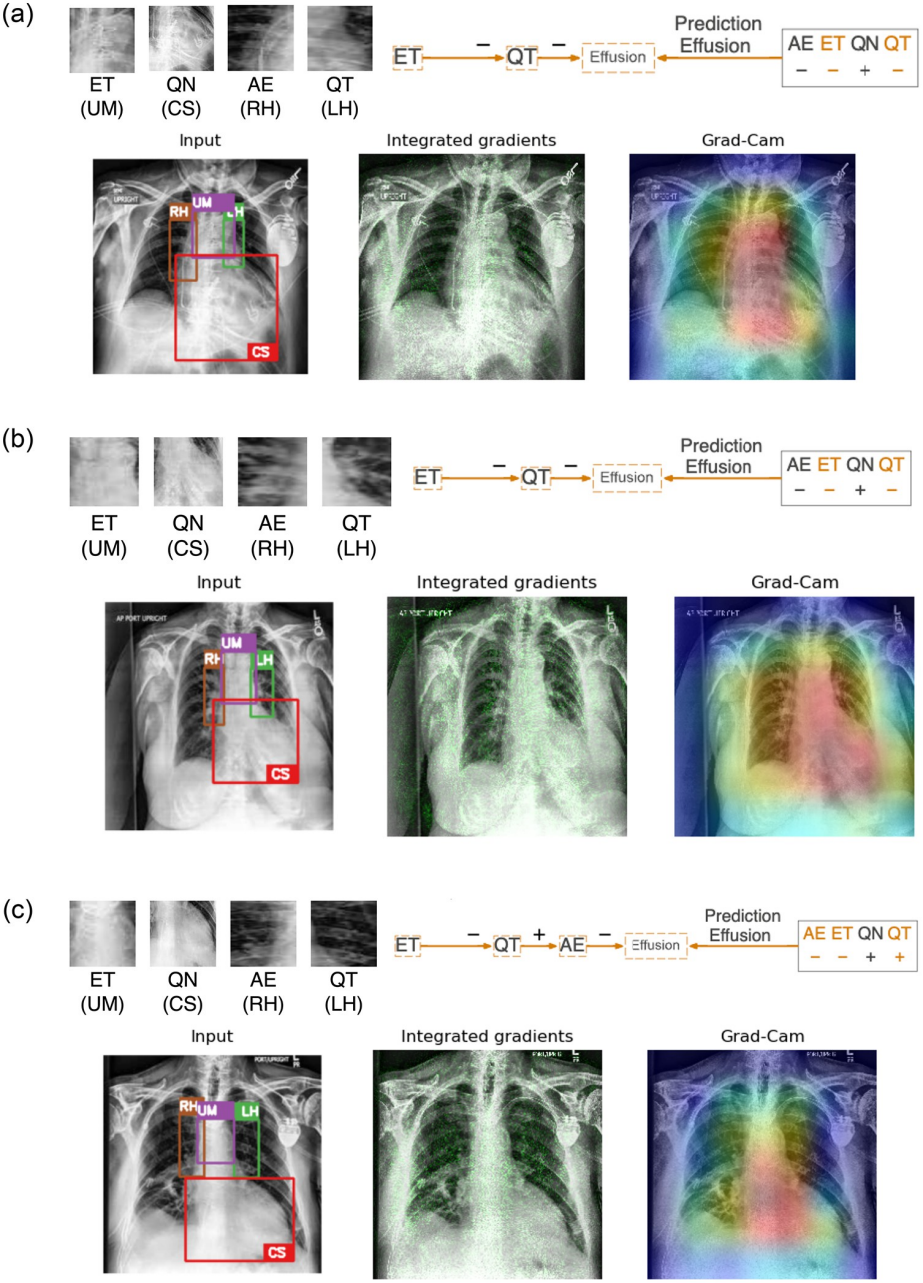
Three representative examples of applying neural-symbolic rules on images with pleural effusion in the training images. (a) The prediction is attributed to an obscured Upper Mediastinum (in purple box with literal ¬*ET*) and the Left Hilar appears to be obscured (in green box with literal ¬*QT*). (b) The prediction in Example #2 can be reasoned on the homogeneous texture at the Upper Mediastinum (literal ¬*ET*) and hazy Left Hilar (literal ¬*QT*). (c) Example #3 illustrates a case where the Upper Mediastinum has an homogeneous texture (¬*ET*) and the prediction utilized the clear visibility of structure at the Left Hilar (literal *QT*) but hazy Right Hilar (literal ¬*AE*). Integrated gradient and Grad-CAM highlight the significance of the mediastinum (in green and red respectively) without further details and reasoning on the predictions of pleural effusion.

Local explanation can also be applied to gain insights into the decision-making process of the clinically relevant rule set (see Fig 3b) used for the classification of the individual X-ray images as either *healthy* or with *pleural effusion*. Representative examples of this rule set on the training set are illustrated in Figs 8 and 9. These examples are presented with comparative visualizations generated through established interpretation methods such as Integrated Gradient [4] and Grad-CAM [5].

As part of the symbolic rule extraction (refer to Section 4 for details), L1-norm value for each individual image is calculated and compared to a user-defined threshold. This comparison determines whether a kernel yields a positive literal (if L1-norm value is above the threshold) or a negative literal otherwise. As an illustrative example using kernel norm plot for kernel QN (see Fig 4a), images with L1-norm values above the threshold (in red) yield positive literals (*QN*), while those below the threshold result in negative literals (¬*QN*). Notably, positive literals (*QN*) tend to affiliate with images labeled as having pleural effusion, exhibiting obscurity (or ‘silhouette sign’) at the Cardiac Silhouette region. Similarly when referring to the kernel norm plots for ET (S9 Fig), QT (S4(a) Fig) and AE (S5(a) Fig), images labeled as pleural effusion are generally associated with negative literals, ¬*ET*, ¬*QT* and ¬*AE* respectively. These negative literals can be characterized by their corresponding radiomics features which tend to indicate a more homogeneous texture reflecting obscurity/reduced visibility of the underlying structures in the regions.

In Fig 8, two examples (a & b) are correctly classified as ‘healthy’ based on the use of kernel ET (Upper Mediastinum) and QN (Cardiac Silhouette) in the rule. Examination of the input image and close-ups of the relevant kernels (ET, QN, AE, QT) reveals a clear Upper Mediastinum region (highlighted in the purple box) displaying the visual presence of the carina branching into the left and right bronchi, leading to a positive ET literal. Simultaneously, the absence of silhouette sign in the Cardiac Silhouette region (highlighted in the red box), characterized by clear lung air space and the left ventricle border, results in a negative QN literal (i.e. ¬*QN*). For the hilar regions represented by kernel AE (Right Hilar—highlighted in brown) and kernel QT (Left Hilar—highlighted in green) where the L1-Norm values of these kernels are highly correlated with Gray Level Non-Uniformity (GLNU) at these regions, Example #1 shows clear visibility of the bronchus and pulmonary arteries alongside visible rib bones resulting in AE and QT being positive literals. In contrast, Example #2 exhibits reduced visibility of the bronchus and pulmonary arteries, as well as haziness in the Left Hilar (QT) resulting both AE and QT being negative literals. These kernels were however not used in the rules for classifying the specific example images.

Example #3 (Fig 8c) provides another illustration of a healthy prediction where the Upper Mediastinum region has a more homogeneous texture yielding a negative ET literal. In this example, the rule incorporates information from kernels representing the hilar regions (i.e. AE and QT) which exhibit clear hilar regions with visible arteries and rib bones resulting in both kernels to form positive literals. It is worth noting that kernel QN is not needed as a literal in the rule despite the negativity of the literal corresponds typically to a healthy Cardiac Silhouette region.

In comparison with the other interpretation methods that only highlight the most important pixels and activation maps through the gradient retracing to the input images, our approach is capable of providing descriptive explanation on how the features at different anatomical regions constitute to the prediction outcome. The other methods primarily emphasize the significance of the cardiac silhouette region, extending to the upper mediastinum and the hilar regions, without providing further details and reasoning behind the predictions from these anatomical regions.

To extend this neural-symbolic approach to cases with pleural effusion, local explanations are presented in Fig 9 on examples that are classified as having pleural effusion. Fig 9a illustrates a case where the Upper Mediastinum and the Cardiac Silhouette region are notably obscured yielding literals ¬*ET* and *QN* respectively. The obscurity also extended to the Left and Right Hilar regions leading to both kernels resulting as negative literals, ¬*QT* and ¬*AE*. The rule relies only on the literals from kernel ET and QT even though the literals from kernel QN and AE also relate typically to cases with pleural effusion.

Fig 9b shows the case where the Upper Mediastinum (kernel ET) has a homogeneous texture with no visibility at the carina, resulting in a negative literal ¬*ET*. Additionally, the Left and Right Hilar appear hazy yielding them negative literals, ¬*QT* and ¬*AE*. The Cardiac Silhouette region is significantly obscured leading to a positive literal QN. However, this prediction again only requires literals from kernel ET and QT.

In Fig 9c, the Upper Mediastinum also has a homogeneous texture leading to a negative literal (¬*ET*). The Left Hilar (kernel QT) exhibits clear visibility of the structure while the Right Hilar (kernel AE) appears hazy as observed in the close-up views resulting in a positive literal *QT* and a negative literal ¬*AE*. These literals have led to the prediction of ‘pleural effusion’ in this case. The Cardiac Silhouette region is not included in the rule but appears obscured leading to a positive literal *QN*, normally related to Cardiac Silhouette regions with pleural effusion.

In all cases of pleural effusion, integrated gradient and Grad-CAM only highlight the importance of the mediastinum and neighboring regions without providing details on how these highlighted regions (shown in green and in red respectively) contribute to the classification outcome as previously discussed.

**Fig 10:**
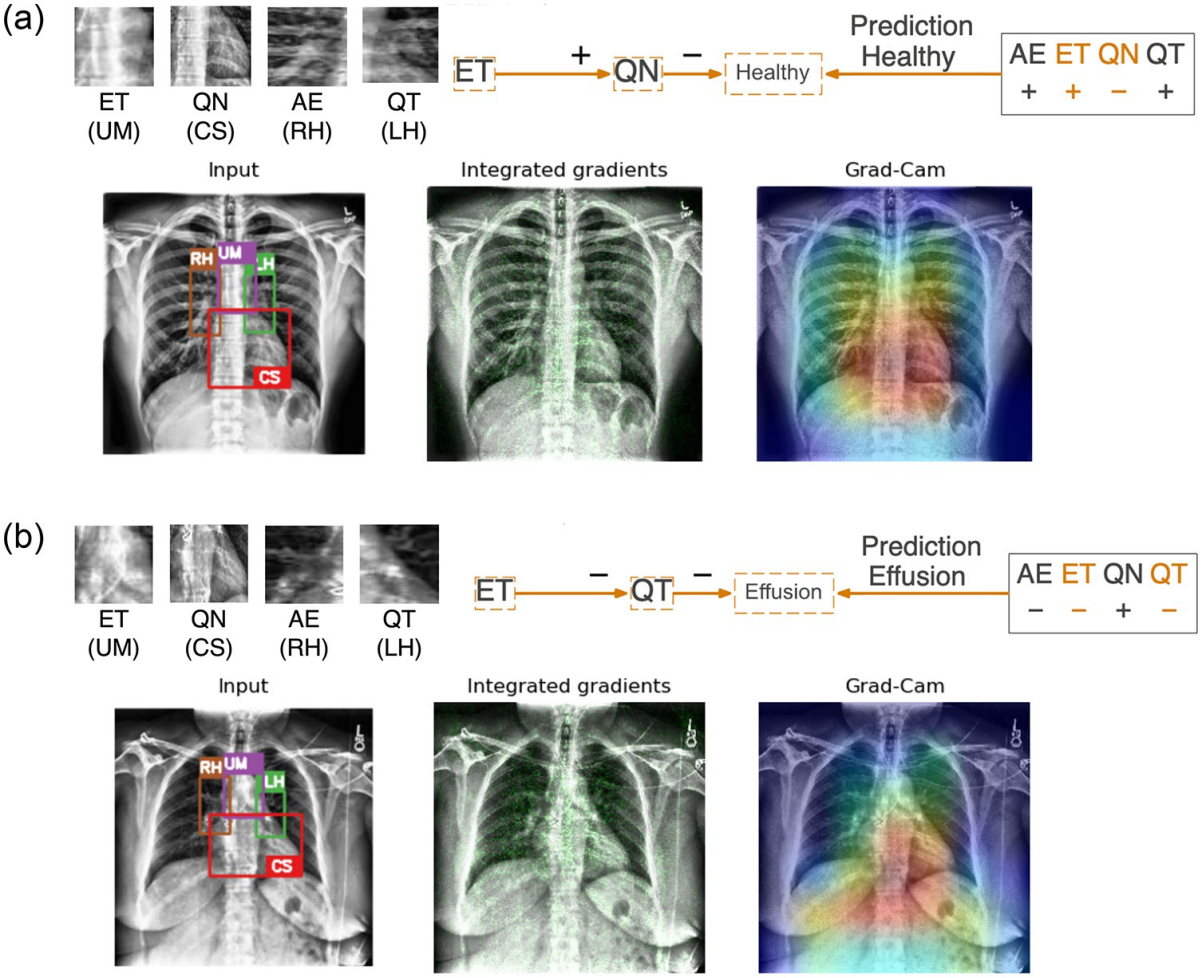
Two representative examples applying neural-symbolic rules on healthy images in the validation images. (a) A correct prediction is attributed to a clear Upper Mediastinum (in purple box with literal *ET*) and the absence of obscurity at the Cardiac Silhouette (in red box with literal ¬*QN*). (b) A false positive prediction can be reasoned due to reduced contrast of the carina due to whiteness at the Upper Mediastinum (literal ¬*ET*) and lower visibility of the structure (e.g. arteries and rib bones) at the Left Hilar (literal ¬*QT*). Integrated gradient and Grad-CAM highlight the significance of the mediastinum (in green and red respectively) without details and reasoning on both predictions.

As illustrated with the training images, our neural symbolic approach for model explanation has demonstrated clear advantage in providing relevant details on the reasoning of the predictions. We also extend the evaluation to other representative validation images. Fig 10 presents two cases: (a) a true classification of a healthy image and (b) a false positive case where an image labeled as ‘healthy’ is wrongly classified as having pleural effusion. For the case in Fig 10a, the correct prediction can be attributed to a clear Upper Mediastinum displaying the visible branching at the carina (with a positive literal *ET*) and the absence of obscurity, commonly referred as ‘silhouette sign’, at the Cardiac Silhouette region (with a negative literal ¬*QN*). Additionally, the hilar regions show clear visibility of arteries and rib bones resulting in positive literals AE and QT. In contrast, whiteness around the carina may have reduced its contrast in Fig 10b at the Upper Mediastinum region leading to a negative literal ¬*ET*. Obscurity at the right ventricle has also led to the Cardiac Silhouette region yielding a positive literal *QN*. Reduced visibility of structure such as arteries and rib bones at the hilar region are also observed when compared to Fig 10a leading to negative literals ¬*AE* and ¬*QT*). While it might not be reflected based on radiomics features (e.g. Gray Level Non-Uniformity), it can also be visually identified that white anomalies can be found at the hilar regions in the close-up views. Based on these information, the false prediction relies on a rule derived from the reduced visibility at the Upper Mediastinum and the Left Hilar regions can be expected from the reasoning.

**Fig 11:**
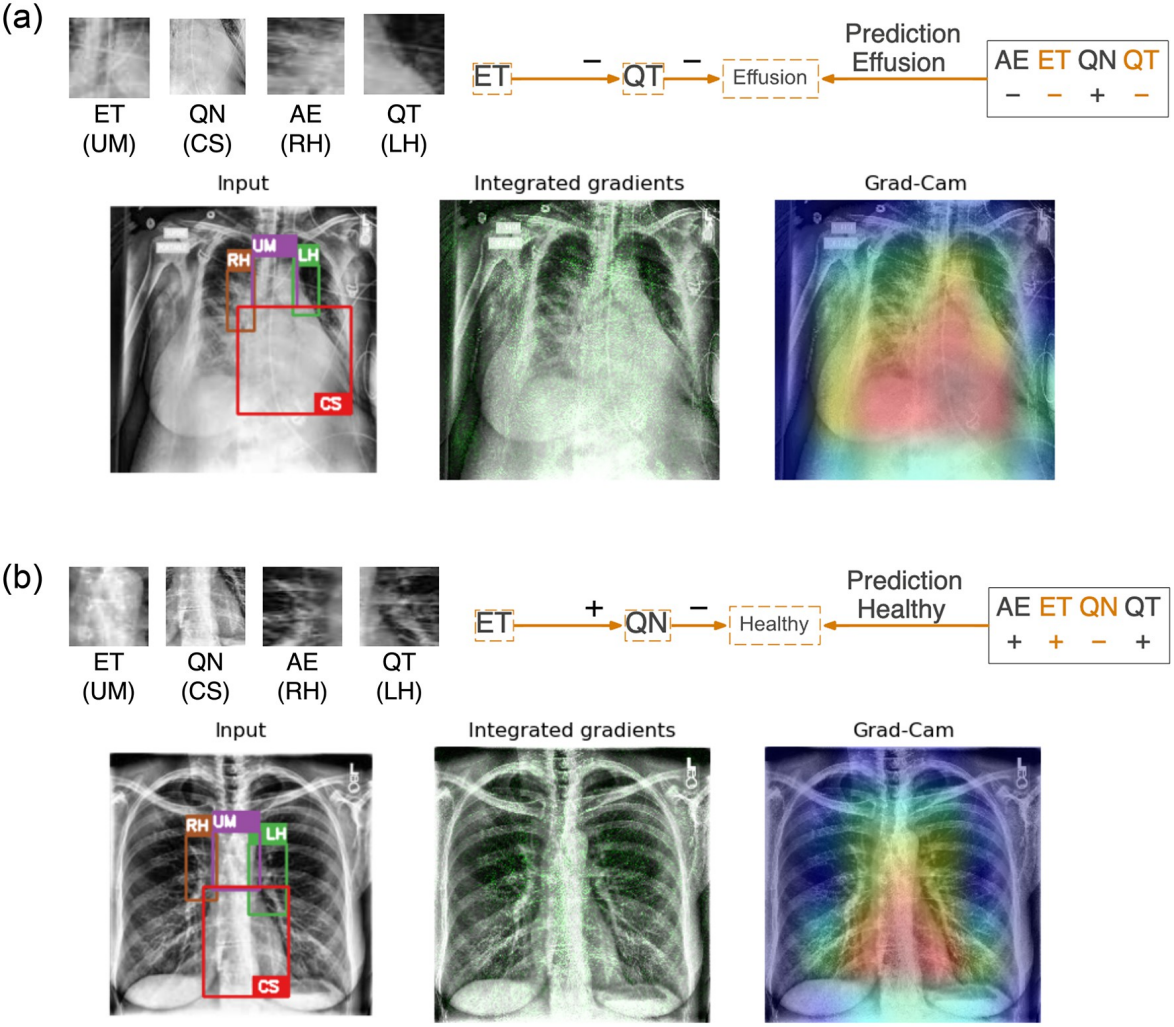
Two representative examples applying neural-symbolic rules on images labeled as pleural effusion in the validation images. (a) A correct prediction is attributed to obscurity at the Upper Mediastinum (in purple box with literal ¬*ET*) and the Left Hilar (in green box with literal ¬*QT*). (b) A false negative prediction can be reasoned due to clear visibility of the carina at the Upper Mediastinum (literal *ET*) and absence of obscurity at the Cardiac Silhouette (literal ¬*QN*). Integrated gradient and Grad-CAM highlight the significance of the mediastinum (in green and red respectively) without details and reasoning on both predictions.

Similarly, representative examples of validation images for pleural effusion are illustrated in Fig 11. Fig 11a shows a true classification for having pleural effusion. Noticeable obscurity can be observed at the Upper Mediastinum and Cardiac Silhouette yielding literals of ¬*ET* and *QN* respectively. The Right Hilar is also obscured with haziness and reduced visibility in structure at the Left Hilar leading to negative literals ¬*AE* and ¬*QT*. The prediction in this case relies solely on the information from the Upper Mediastinum and the Left Hilar, despite relevant information at the Cardiac Silhouette and the Right Hilar also reflecting on the presence of pleural effusion. In contrast, the false classification in Fig 11b aligns more closely with literals typically associated with a healthy image. For example, the Upper Mediastinum is considered to be distinctly visible at the carina (with literal *ET*), the ventricle can also be clearly identified at the Cardiac Silhouette region (with literal ¬*QN*), and both Left and Right Hilar regions are notably clear with visible arteries and rib bones. The false prediction can be reasoned through the literals, *ET* and ¬*QN*, relating to the Upper Mediastinum and the Cardiac Silhouette in the rule.

The integrated gradient and Grad-CAM only highlight the mediastinum which no doubt plays a crucial role in this classification task. These methods however do not provide details and reasoning on the prediction outcome. We regard our approach to be more valuable in understanding the generation of the predictions during model training and allowing better identification on the cause of false predictions based on the underlying reasoning when applied to other images. The reasoning of false predictions can subsequently be passed on to the domain experts for more thorough human evaluation to ensure an accurate diagnosis.

## 7 Significance of anatomically relevant kernels on model performance

**Fig 12:**
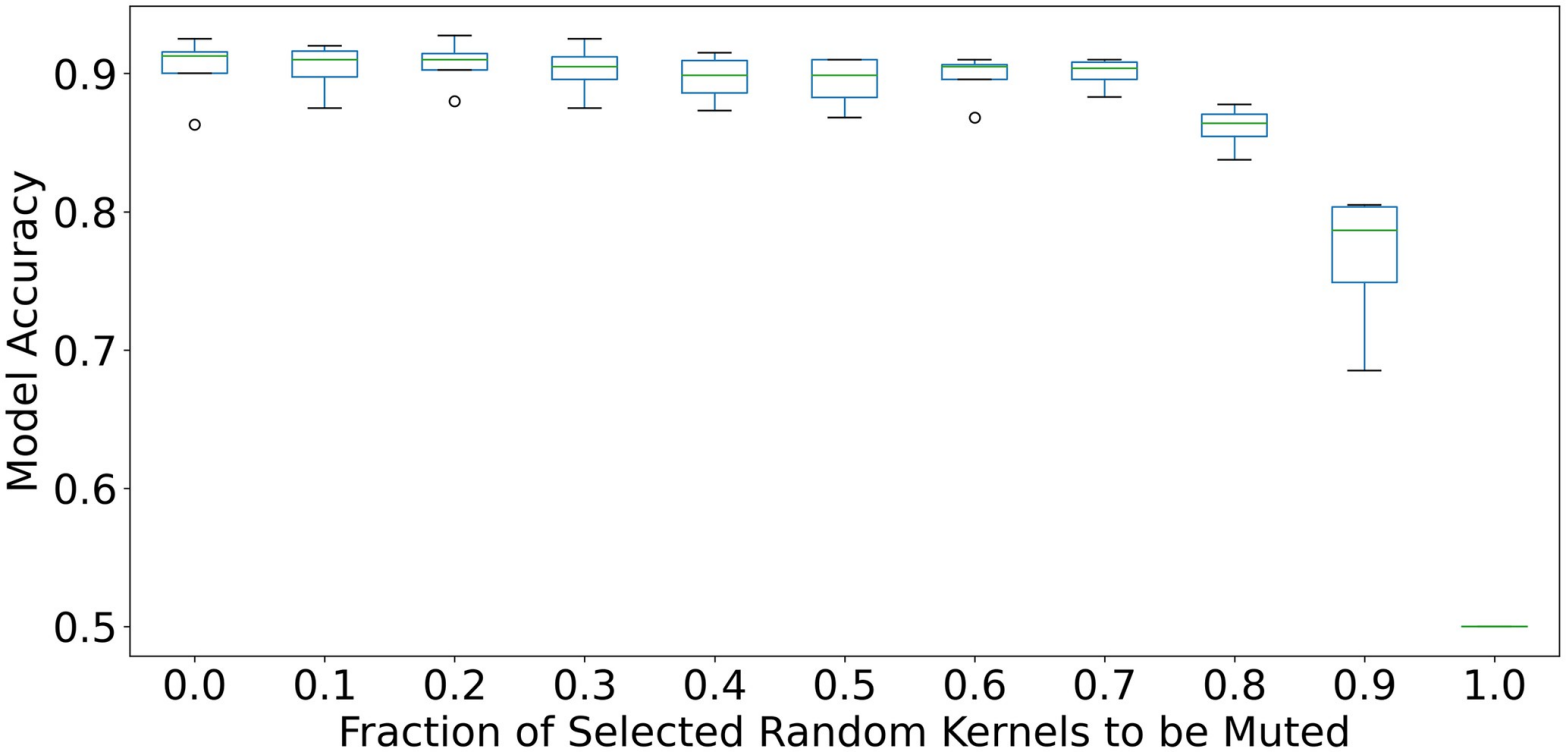
Evaluation of model accuracy degradation by muting a random selection of an increasing number of kernels at 10% intervals when applied to trained CNN models on pleural effusion classification (Originated from [13]).

In a recent work [13], it was found that a CNN model required significantly fewer kernels to maintain the accuracy of a binary classification. Fig 12 shows that up to 70% of the kernels in a trained VGG-16 model for pleural effusion classification could be randomly muted (demonstrated over 20 repeated runs) without affecting model performance.

**Fig 13:**
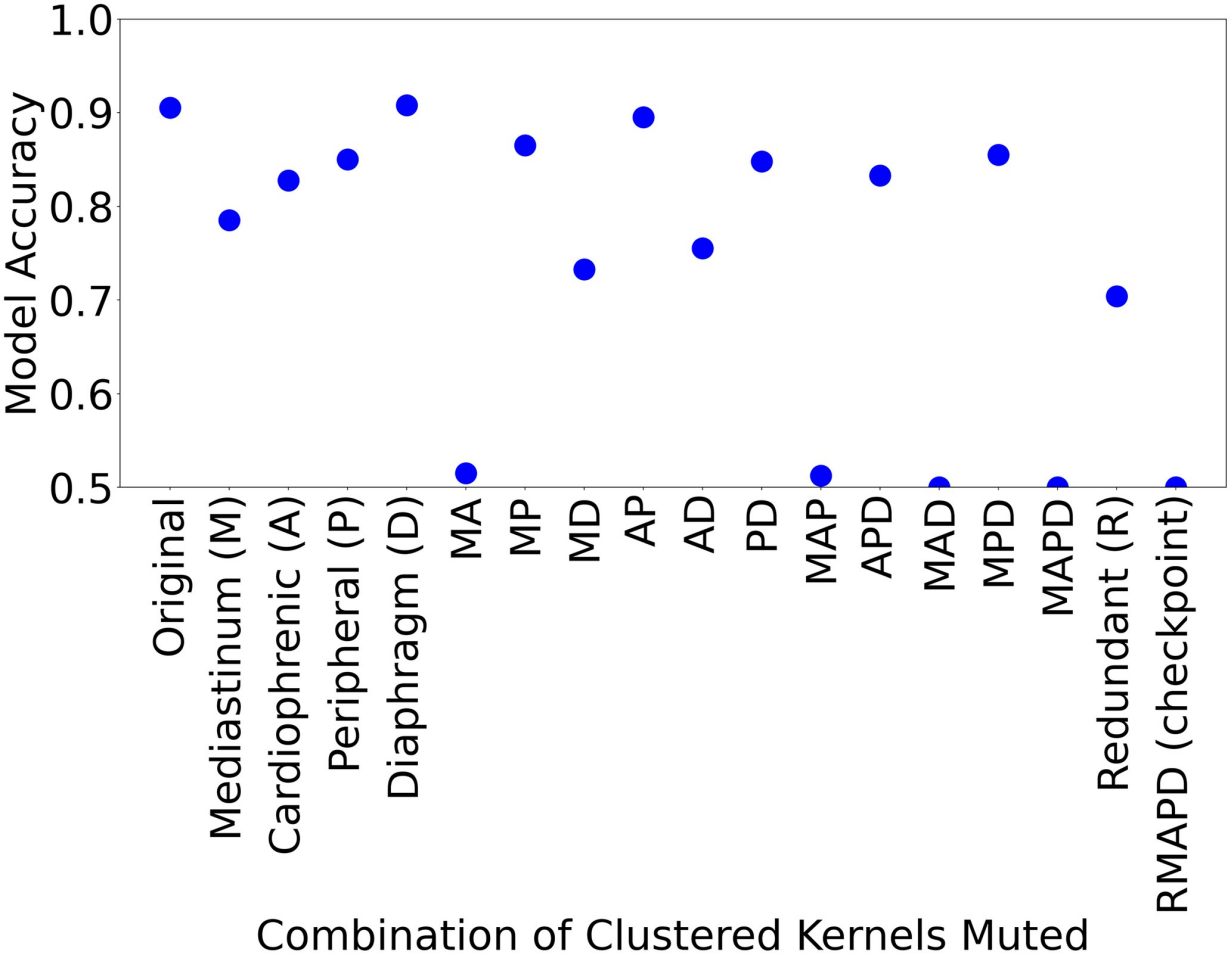
Evaluation of average model accuracy by muting kernels in anatomically relevant clusters (e.g. Mediastinum (M), Cardiophrenic Angle (A)) and its combinations (e.g. Mediastinum and Caradiophrenic Angle (MA), Mediastinum and Lung Peripheral (MP)) over five trained CNN models for pleural effusion (Originated from [13]).

In addition, it was found that the presence of kernels deemed to be meaningful to the task at hand was important to maintain the performance of the model compared to kernels that were conceptually unclear (see Fig 13). For example, muting only kernels of the Diaphragm (D), or kernels from both the Cardiophrenic Angle and the Peripheral (AP) would not result in a significant drop in model accuracy, by contrast with muting kernels from the Mediastinum and Cardiophrenic Angle together (MA).

The work in this paper extends these preliminary findings to establish the significance of domain-relevant kernels on model performance. As explained in Section 5.4, kernel fingerprints were replaced with more appropriately defined anatomical regions, as a better representation for the attention regions of a CNN trained on frontal chest X-rays.

**Fig 14:**
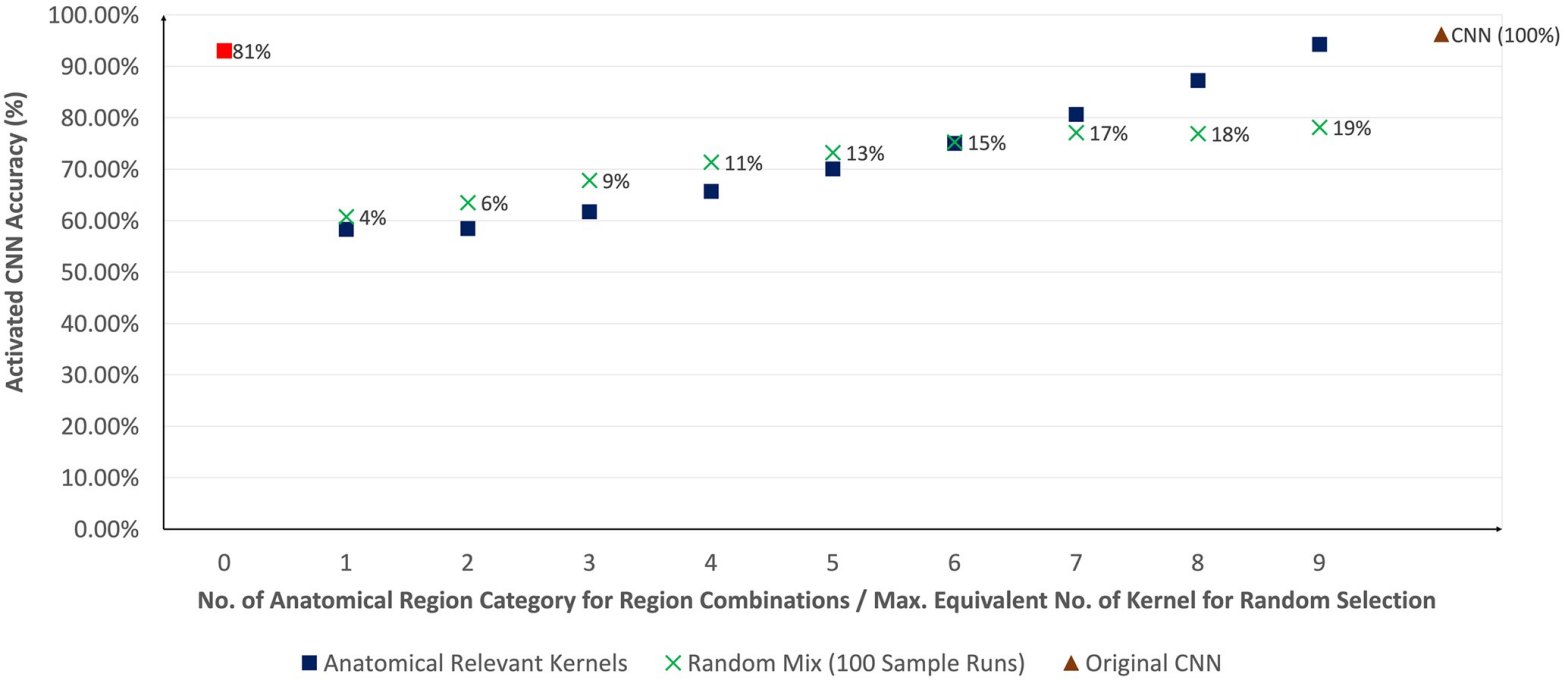
Evaluation of average model accuracy with the comparison between incremental activation of anatomical relevant kernel groups (blue square) and an equivalent random mix of uninterpretable kernels over 100 sample runs (green cross). An additional run of activating all uninterpretable kernels alone (red square) is also displayed. The percentage in each data point reflects the maximum percentage of kernels being activated. The evaluation shows that increasing anatomical relevant kernels improves model accuracy more than a random selection. In comparison, the accuracy of the original CNN (i.e. all kernels activated in brown triangle) is 96.2%.

Fig 14 shows an expected gain in model accuracy as more kernels are activated (i.e. from kernels of a single group of an anatomical region to kernels from all 9 anatomical regions as described in Section 5.4). When kernels from all anatomical regions were activated even at only 19% of all kernels, the model was capable of achieving an accuracy of 94.3% compared to the original CNN model with 96.2%. Activating only all the remaining kernels (i.e. 81% of all kernels) that were deemed to be uninterpretable achieved an accuracy of 93.0%. It should also be noted that the model improvement by using relevant kernels was higher once sufficient information was extracted from the kernels (i.e. activating kernels in more than 6 anatomical regions). The model accuracy of activating a random mix of uninterpretable kernels was also notably higher than 50% (random guess in a binary classification) and it plateaued at approximately 78%. All these findings imply that relevant information for model prediction remained embedded within the uninterpretable kernels. This should lead to a direction for further investigation with better medical understanding to define the specific conceptual meaning of these kernels.

**Fig 15:**
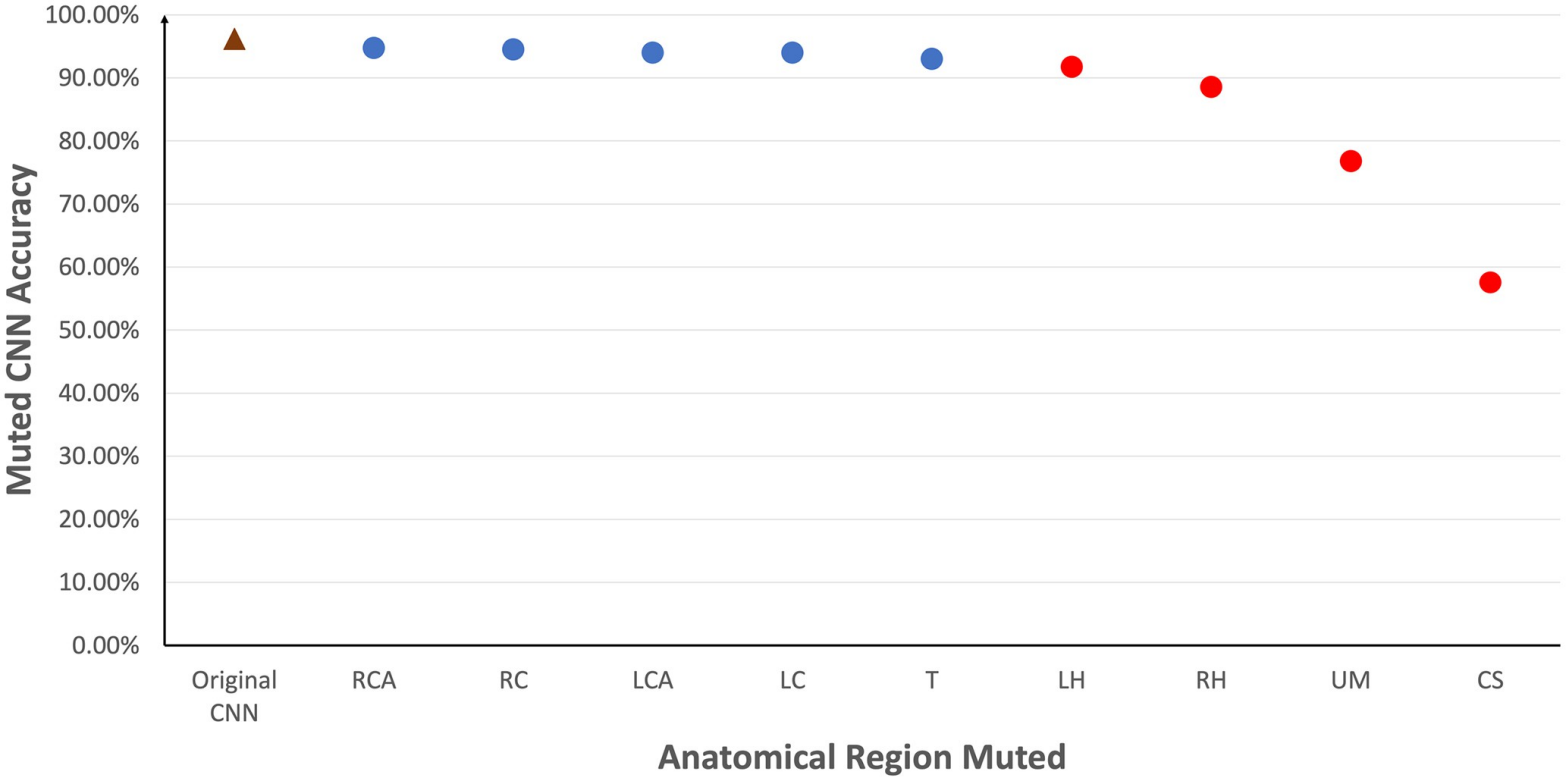
Evaluation of the effect of muting kernels that are uninterpretable and a single anatomical region on the model accuracy. The result (in red) shows that muting kernels from Cardiac Silhouette (CS), Upper Mediastinum (UM), Right Hilar (RH) and Left Hilar (LH) has shown a significant drop in accuracy (i.e. a decline of at least 4.5% from the original CNN accuracy of 96.2%).

**Fig 16:**
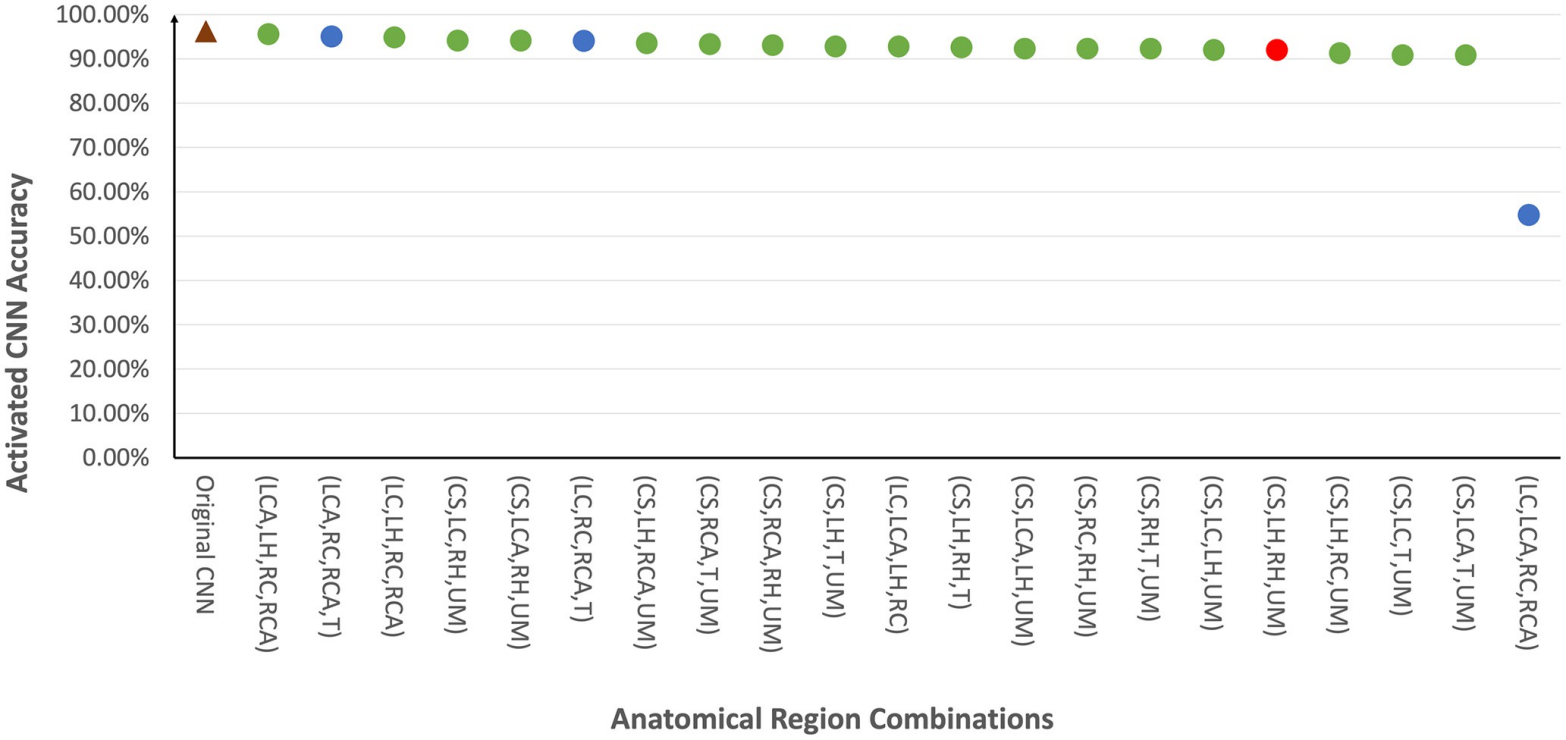
Top 20 Combinations of kernels from 4 anatomical regions with the highest model accuracy. Cardiac Silhouette (CS), Upper Mediastinum (UM), Right Hilar (RH) and Left Hilar (LH) are found in majority of these combinations (in green dots) especially with Cardiac Silhouette (CS) and Upper Mediastinum (UM) together. The combination of (CS,LH,RH,UM) in red represents the anatomical regions used in the clinically relevant decision tree in Fig 3b an accuracy of 92.0%. Trachea (T) is also frequently observed among the highest performing combinations beyond those 4 regions used in the decision tree. On the other hand, the accuracy from a combination that activates solely on the clavicle bones (LC, RC) and the costophrenic angles (LCA, RCA) is only 54.8%. These results can also be compared with the accuracy of the original CNN (i.e. all kernels activated in brown triangle) of 96.2%.

Given that it was observed that kernels representing specific anatomical regions may impact model accuracy, Fig 15 evaluates the effect on model accuracy by muting all uninterpretable kernels as well as kernels from one anatomical region. It was observed that muting kernels from Cardiac Silhouette (CS), Upper Mediastinum (UM), Right Hilar (RH) and Left Hilar (LH) led to a decrease in accuracy of at least 4.5%. This coincided with the kernels used in the clinically relevant decision tree in Section 6. In addition, it was also found that these 4 regions are present in the majority of the top 20 highest performing combinations of activated kernels, especially with Cardiac Silhouette (CS) and Upper Mediastinum (UM) together. Specifically, the combination (CS,LH,RH,UM) used in the decision tree yields an accuracy of 92.0%. Trachea (T) also appeared frequently among the highest performing combinations beyond those anatomical regions used in the decision tree. On the other hand, a combination using solely clavicle bones (LC,RC) and the costophrenic angles (LCA,RCA) would yield an accuracy of only 54.8%. It should be noted that the top performing combinations maintained accuracy close to that of the original CNN despite using no more than 11% of the kernels in the original CNN model(see Fig 16).

## 8 Conclusion and future work

This work has presented an interactive framework for model explanation that maps a complex CNN model onto interpretable symbolic rules represented as a decision tree. Automatic anatomical region localisation was applied to attach meaning to the kernel activations. Kernel norm plots assisted a medical expert in interpreting the constituent kernels in order to derive a concept represented by each kernel. A graphical user interface enabled interaction and intervention by an expert to generate more clinically relevant explanations by modifying the extracted decision tree. This work was demonstrated to provide meaningful kernel descriptions using radiomics features and evaluating their relevance to the prediction outcome.

Based on the empirical evidence presented in this work, an initial set of rules consisting of irrelevant and uninterpretable kernels was replaced with alternatives consisting of clinically explainable kernels. Medical relevance could therefore be established from the anatomical regions that these kernels represent [41, 44, 45]. Supporting radiomics feature analysis also discovered that these kernels were a quantifiable estimation of the changes in pixel intensity relative to its spatial neighbourhood (e.g. visibility of the ventricle in the cardiac silhouette region). This exemplified a similar approach to how a radiologist may view an X-ray image by identifying visual characteristics on relevant anatomical regions. The modification was also found to have a negligible deterioration in the predictive performance of the model.

Forming a rule set using irrelevant kernels had been demonstrated to produce poor performance. This finding was consistent with previous research [13]. This also implied that these kernels could be disregarded for the task until appropriate conceptual descriptions could be defined. It also indicated that the model could be pruned, with the binary classification task to distinguish pleural effusion from healthy cases only requiring a small number of kernels for good prediction. On the same note, this also demonstrated the limitations of such model as a comprehensive diagnostic system for all respiratory diseases (e.g. pleural effusion vs tuberculosis where regions in the upper lung zones may become important [46]). Where data availability permits, this opens the opportunity to extend this work to multi-class classification tasks for various respiratory diseases.

The association between supporting radiomics features and kernel norms indicated that the kernels were indeed extracting texture information from pixels across convolutional layers. While it was possible to observe changes through the range of the best-suited radiomics feature, distinguishing the effect between similarly represented radiomics features (e.g. Gray Level Non-Uniformity vs Pixel Intensity Root Mean Squared Values) remained challenging. It will be valuable to conduct additional research to uncover the finer details that may enhance the current progress in kernel concept definition.

In the future when new medical knowledge is discovered, current uninterpretable kernels should be reviewed so that concepts can be assigned appropriately. This may also apply for kernels that are currently considered to be irrelevant (i.e. right clavicle) as in the case of the illustrative example where a patient’s positioning at the time that the X-ray is taken may become important. Additionally, future research can be conducted on transferring knowledge from pleural effusion kernels to other respiratory diseases.

## Supporting information

S1 Appendix This appendix provides a quantitative assessment of the segmentation model performance, which uses the YOLOv5x architecture as referenced in [40], for anatomical region localisation. Specifically, it presents the superiority of the model in the designated task but also highlights the limitation for regions of the Hilar and Costophrenic Angle given the similarity of these regions at the left and right lungs. Since all the images used in this study are frontal, the consistent positioning of the anatomical regions simplifies the process of identifying any missing or incorrectly detected regions to enable manual corrections.
(PDF)

S2 Appendix This appendix presents the findings from our investigation into the impact of alternative kernels for the same anatomical region when they are applied in symbolic rules, along with the corresponding radiomics association as concept description. It also provides further details, which complements the discussion in the main text, regarding how radiomics features can be used to translate kernels into clinically relevant concepts. This radiomics association is particularly focused on the regions of Cardiac Silhouette, Upper Mediastinum and the Hilars where they are found to be essential for clinically relevant rule formation.
(PDF)

S1 Fig Evaluation of performance on YOLOv5x model for anatomical region localization using NIH dataset. (a) F1-Score plot for different anatomical regions across confidence thresholds. It shows that the hilar and the costophrenic angle regions are the most challenging. (b) Confusion Matrix shows that the labeling of the anatomical regions are highly accurate.
(tif)

S2 Fig Samples of anatomical region localization using YOLOv5x model for plain chest X-rays of patients with different severity in pleural effusion. Any missing regions (e.g. Costophrenic Angles) have been manually added prior to the radiomics analysis.
(tif)

S3 Fig Alternative rule set constructed by changing the kernel relating to (a) Left Hilar (from QT to DM) and (b) Right Hilar (from AE to MH).
(tif)

S4 Fig The kernel norm plot (L1-norm values) for (a) kernel QT and (b) Kernel DM. The first 200 data points are labelled as healthy and the next 200 as pleural effusion in the ground truth. A threshold value (red line) separates positive literals (above the line) and negative literals.
(tif)

S5 Fig The kernel norm plot (L1-norm values) for (a) kernel AE and (b) Kernel MH. The first 200 data points are labelled as healthy and the next 200 as pleural effusion in the ground truth. A threshold value (red line) separates positive literals (above the line) and negative literals.
(tif)

S6 Fig The kernel norm plot (L1-norm values) for (a) kernel AE and (b) Kernel MH. The first 200 data points are labelled as healthy and the next 200 as pleural effusion in the ground truth. A threshold value (red line) separates positive literals (above the line) and negative literals.
(tif)

S7 Fig Correlation between Run Length Gray Level Non-Uniformity (GLRLM) with L1-Norms for (a) Kernel QT and (b) Kernel DM. Sub-figure (c) shows images of the Left Hilar region sorted row-wise from highest Run Length Gray Level Non-Uniformity (GLRLM) (top left) to lowest Run Length Gray Level Non-Uniformity (GLRLM) (bottom right). Those images with *healthy* as ground truth are outlined green while those with *pleural effusion* are outlined red.
(tif)

S8 Fig Correlation between (a) between Run Length Gray Level Non-Uniformity (GLRLM) and L1-Norms for Kernel AE, and (b) between First Order Pixel Intensity Root Mean Squared and L1-Norms for Kernel MH. Sub-figure (c & d) shows images of the Right Hilar region sorted by Run Length Gray Level Non-Uniformity (GLRLM) and Pixel Intensity Root Mean Squared row-wise respectively from highest value (top left) to lowest value (bottom right). Those images with *healthy* as ground truth are outlined green while those with *pleural effusion* are outlined red.
(tif)

S9 Fig The kernel norm plot (L1-norm values) for kernel ET. The first 200 data points are labeled as healthy and the next 200 as pleural effusion in the ground truth. A threshold value (red line) separates positive literals (above the line) and negative literals.
(tif)

S10 Fig (a) A postive correlation between between First Order Mean Absolute Deviation (FOMAD) and L1-Norms for Kernel ET. Sub-figure (b) shows images of the Upper Mediastinum region sorted by First Order Mean Absolute Deviation (FOMAD) from highest value (top left) to lowest value (bottom right). Those images with *healthy* as ground truth are outlined green while those with *pleural effusion* are outlined red.
(tif)
