# Supplementary material for: From explanation to intervention: Interactive knowledge extraction from Convolutional Neural Networks used in radiology
Source: PLoS One. 2024 Apr 10;19(4):e0293967. doi: 10.1371/journal.pone.0293967 (PMC11006149; doi:10.1371/journal.pone.0293967)
Supplement: S1 Appendix — Specifically, it presents the superiority of the model in the designated task but also highlights the limitation for regions of the Hilar and Costophrenic Angle given the similarity of these regions at the left and right lungs. Since all the images used in this study are frontal, the consistent positioning of the anatomical regions simplifies the process of identifying any missing or incorrectly detected regions to enable manual corrections. (PDF) [file pone.0293967.s001.pdf]

Supporting Information

Appendix S1 Supplementary Materials on Anatomical Region Localisation

This section presents a quantitative evaluation of the segmentation model using a YOLOv5x architecture [40] (see S1 Fig). Except for the Hilar and Costophrenic Angle regions, the F1 score for all other regions across a wide range of confidence thresholds is high (i.e. > 0.8). Those inferior regions were very similar between regions in the left and right lungs. In addition, the hilars were also very similar to the rest of the lung space, particularly along the vertical axis. The confusion matrix further revealed that the labeling of the detected regions was highly accurate. S2 Fig showed some of the raw output from the segmentation model on X-rays of healthy and pleural effusion patients with varying severity.

Due to the fact that all images used in this work were frontal, the relative positioning of the anatomical regions were highly consistent. This facilitates inspection for missing/incorrect localized regions and manual correction where necessary. This proved to be useful in subsequent work evaluating the texture changes (radiomics features) for specific regions observed in the kernel norm plots.

S1 Fig. Evaluation of performance on YOLOv5x model for anatomical region localization using NIH dataset. (a) F1-Score plot for different anatomical regions across confidence thresholds. It shows that the hilar and the costophrenic angle regions are the most challenging. (b) Confusion Matrix shows that the labeling of the anatomical regions are highly accurate.

S2 Fig. Samples of anatomical region localization (a-c) using YOLOv5x model for plain chest X-rays of patients with different severity in pleural effusion. Any missing regions (e.g. Costophrenic Angles) have been manually added prior to the radiomics analysis.

References

40. Jocher et al G. YOLOv5 SOTA Realtime Instance Segmentation; 2022.
