# Supplementary material for: From explanation to intervention: Interactive knowledge extraction from Convolutional Neural Networks used in radiology
Source: PLoS One. 2024 Apr 10;19(4):e0293967. doi: 10.1371/journal.pone.0293967 (PMC11006149; doi:10.1371/journal.pone.0293967)
Supplement: S2 Appendix — It also provides further details, which complements the discussion in the main text, regarding how radiomics features can be used to translate kernels into clinically relevant concepts. This radiomics association is particularly focused on the regions of Cardiac Silhouette, Upper Mediastinum and the Hilars where they are found to be essential for clinically relevant rule formation. (PDF) [file pone.0293967.s002.pdf]

Supporting Information

Appendix S2 Radiomics feature analysis on anatomical regions represented by the chosen kernels

In this section, results on the effect of using alternative kernels of the same anatomical region, as well as the corresponding radiomics findings were presented. S3 Fig showed the change for (a) the Left Hilar (from kernel QT to kernel DM) and (b) the Right Hilar (from kernel AE to kernel MH). A change in the kernel for the Left Hilar resulted in a sensitivity of 92.5% and a specificity of 93.5%, while a change in the kernel for the Right Hilar resulted in a sensitivity of 91.5% and a specificity of 94.5%. The fidelity to the original CNN model remained high at 95.8% in both instances.

The radiomics feature analysis for kernels representing (a) the Cardiac Silhouette (QN and OD), (b) the Left Hilar (QT and DM), and (c) the Right Hilar (AE and MH) were presented in S6 Fig, S7 Fig and S8 Fig respectively. It appeared that the kernel norm values (L1-norms) for both kernels of the Cardiac Silhouette region were correlated with the Joint Entropy (GLCM). As shown in S6 Fig(c), this corresponded to visual observation of the texture at the region where the border between the ventricle and the spine bone became invisible as the L1-norm value increases (i.e. from healthy (high Joint Entropy) to pleural effusion (low Joint Entropy)).

In the Left Hilar region, the correlation between L1-Norm values for kernel QT and DM with Run Length Gray Level Non-Uniformity (GLRLM) was positive. As seen in S4 Fig, the L1-norm values were high in healthy cases for both Kernel QT and DM (i.e. the first 200) and low for the pleural effusion cases (i.e. remaining 200). As a result, it explained the positive correlation observed when compared to the Gray Level Non-Uniformity feature (i.e high for the healthy cases and low for pleural effusion cases). This corresponded to the visual observation that the Left Hilar region becomes opaque as the presence of pleural effusion increases (denoted by the change in L1-norm values).

For the Right Hilar, kernel AE had a positive correlation with the radiomics features to which it was most closely fitted, namely Gray Level Non-Uniformity (GLRLM) (i.e. high L1 norm values and high Gray Level Non-Uniformity for healthy cases and vice versa) while kernel MH had a negative correlation with the First Order Pixel Intensity Root Mean Squared (i.e. high L1-norm values and low Pixel Intensity Root Mean Squared for healthy cases and vice versa) respectively. By observing S8 Fig (c - d), the change in L1-norm values (see S5 Fig) could again be translated to the visual observation of the right hilar, which became more opaque in the presence of pleural effusion.

Lastly, the change in L1-norm values at the Upper Mediastinum for kernel ET (see S9 Fig) can be positively correlated to the First Order Mean Absolute Deviation (FOMAD) (see S10 Fig(a)) that can be translated to a visual observation of a visible branching of the trachea to the bronchus at the carina with high L1-norm values and high Mean Absolute Deviation (top left) and an obscured Upper Mediastinum with more homogeneous texture otherwise (bottom right) (see S10 Fig(b)).

This radiomics analysis had illustrated that the L1-norm values displayed in the kernel norm plot could be used to approximate the change in visual texture in a particular region, simulating how a clinician would examine an X-ray image. However,

the exact difference between visual changes caused by changes in, say, Gray Level Non-Uniformity (GLRLM) and First Order Pixel Intensity Root Mean Squared remains unclear. This paved the way for future research to enhance the concept definition with these finer details through more in-depth analysis.

S3 Fig. Alternative rule set constructed by changing the kernel relating to (a) Left Hilar (from QT to DM) and (b) Right Hilar (from AE to MH).

S4 Fig. The kernel norm plot (L1-norm values) for (a) kernel QT and (b) Kernel DM. The first 200 data points are labelled as *healthy* and the next 200 as *pleural effusion* in the ground truth. A threshold value (red line) separates positive literals (above the line) and negative literals.

S5 Fig. The kernel norm plot (L1-norm values) for (a) kernel AE and (b) Kernel MH. The first 200 data points are labelled as *healthy* and the next 200 as *pleural effusion* in the ground truth. A threshold value (red line) separates positive literals (above the line) and negative literals.

S6 Fig. A negative correlation between Joint Entropy (GLCM) with L1-Norms for (a) Kernel QN and (b) Kernel OD. Sub-figure (c) shows images of the Cardiac Silhouette region sorted row-wise from highest Joint Entropy (top left) to lowest Joint Entropy (bottom right). Those images with *healthy* as ground truth are outlined green while those with *pleural effusion* are outlined red.

S7 Fig. Correlation between Run Length Gray Level Non-Uniformity (GLRLM) with L1-Norms for (a) Kernel QT and (b) Kernel DM. Sub-figure (c) shows images of the Left Hilar region sorted row-wise from highest Run Length Gray Level Non-Uniformity (GLRLM) (top left) to lowest Run Length Gray Level Non-Uniformity (GLRLM) (bottom right). Those images with *healthy* as ground truth are outlined green while those with *pleural effusion* are outlined red.

S8 Fig. Correlation between (a) between Run Length Gray Level Non-Uniformity (GLRLM) and L1-Norms for Kernel AE, and (b) between First Order Pixel Intensity Root Mean Squared and L1-Norms for Kernel MH. Sub-figure (c & d) shows images of the Right Hilar region sorted by Run Length Gray Level Non-Uniformity (GLRLM) and Pixel Intensity Root Mean Squared row-wise respectively from highest value (top left) to lowest value (bottom right). Those images with *healthy* as ground truth are outlined green while those with *pleural effusion* are outlined red.

S9 Fig. The kernel norm plot (L1-norm values) for kernel ET. The first 200 data points are labeled as *healthy* and the next 200 as *pleural effusion* in the ground truth. A threshold value (red line) separates positive literals (above the line) and negative literals.

S10 Fig. (a) A postive correlation between between First Order Mean Absolute Deviation (FOMAD) and L1-Norms for Kernel ET. Sub-figure (b) shows images of the Upper Mediastinum region sorted by First Order Mean Absolute Deviation (FOMAD) from highest value (top left) to lowest value (bottom right). Those images with *healthy* as ground truth are outlined green while those with *pleural effusion* are outlined red.
