# Supplementary figures and images for: From explanation to intervention: Interactive knowledge extraction from Convolutional Neural Networks used in radiology
Source: PLoS One. 2024 Apr 10;19(4):e0293967. doi: 10.1371/journal.pone.0293967 (PMC11006149; doi:10.1371/journal.pone.0293967)

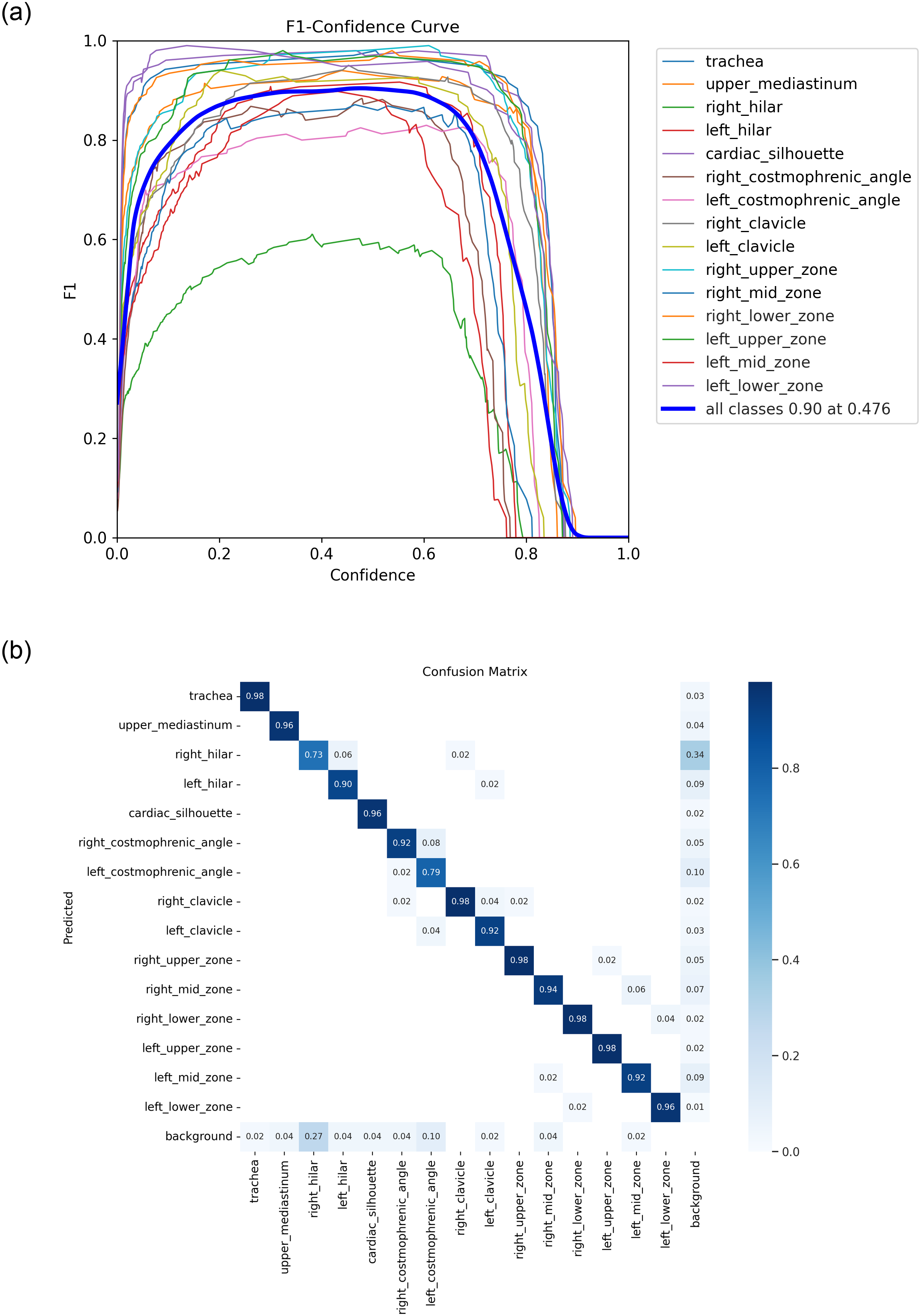

Supplement: S1 Fig — (a) F1-Score plot for different anatomical regions across confidence thresholds. It shows that the hilar and the costophrenic angle regions are the most challenging. (b) Confusion Matrix shows that the labeling of the anatomical regions are highly accurate. (tif) [file pone.0293967.s003.tif]

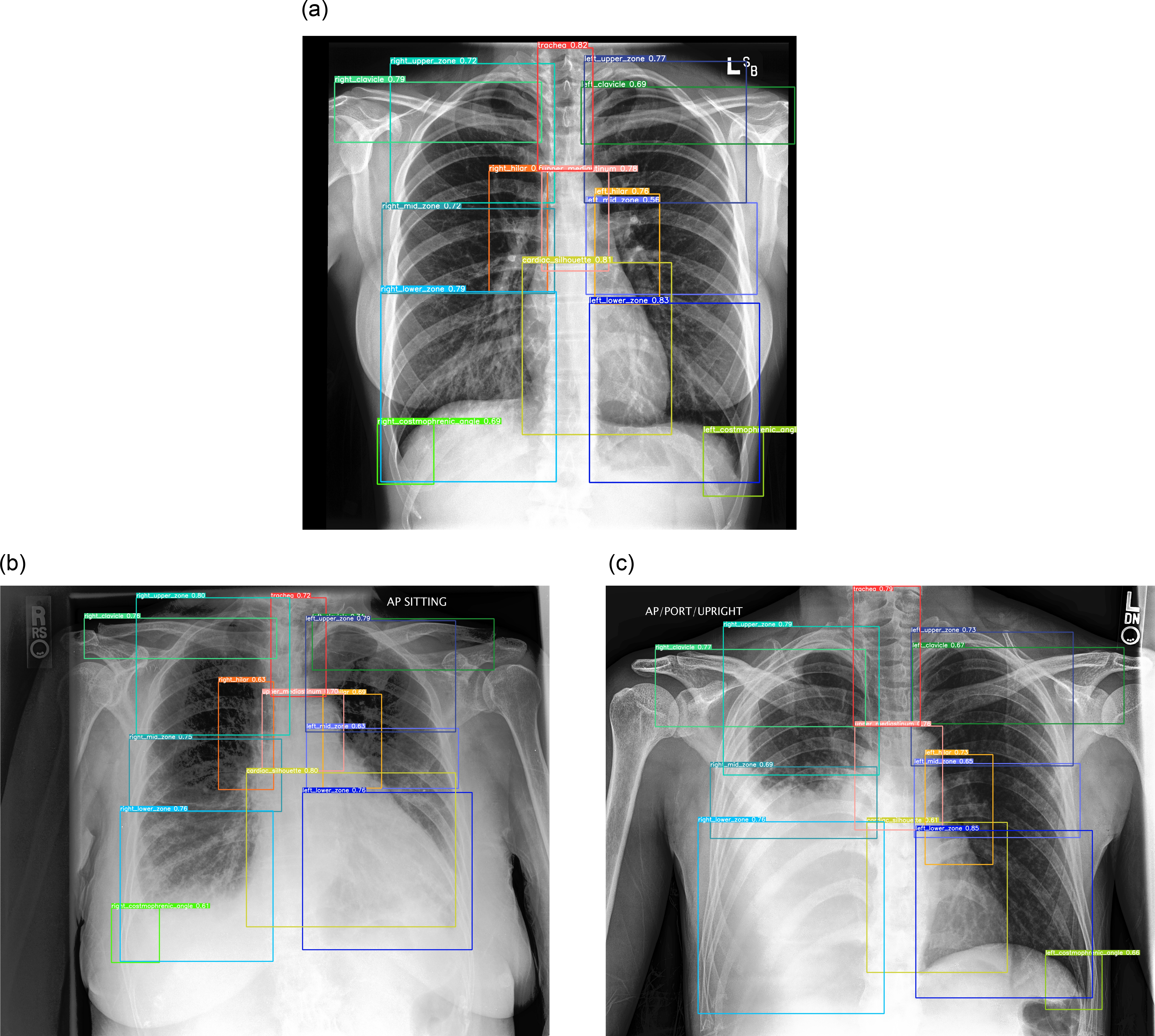

Supplement: S2 Fig — Any missing regions (e.g. Costophrenic Angles) have been manually added prior to the radiomics analysis. (tif) [file pone.0293967.s004.tif]

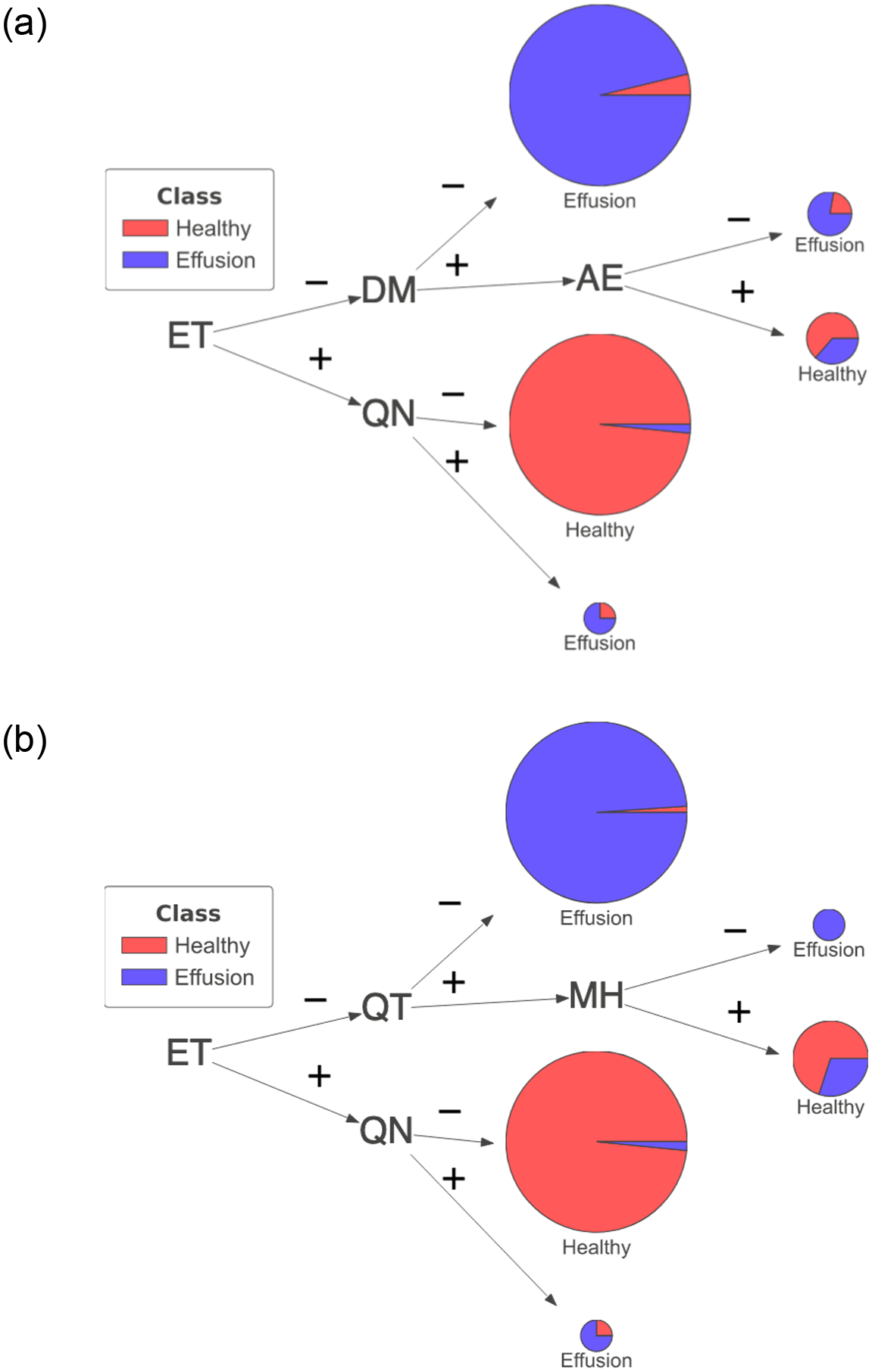

Supplement: S3 Fig — Alternative rule set constructed by changing the kernel relating to (a) Left Hilar (from QT to DM) and (b) Right Hilar (from AE to MH). (tif) [file pone.0293967.s005.tif]

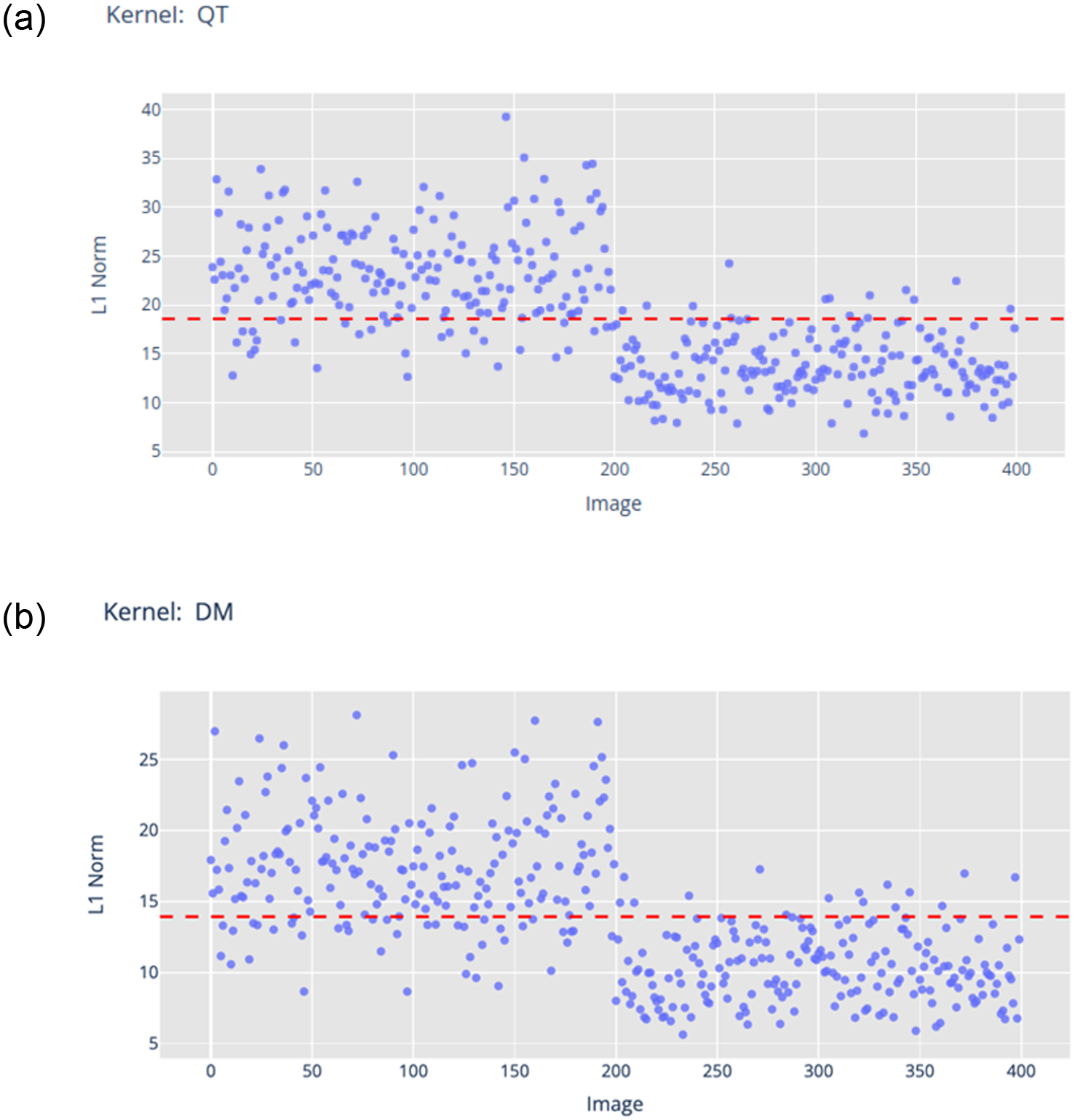

Supplement: S4 Fig — The first 200 data points are labelled as healthy and the next 200 as pleural effusion in the ground truth. A threshold value (red line) separates positive literals (above the line) and negative literals. (tif) [file pone.0293967.s006.tif]

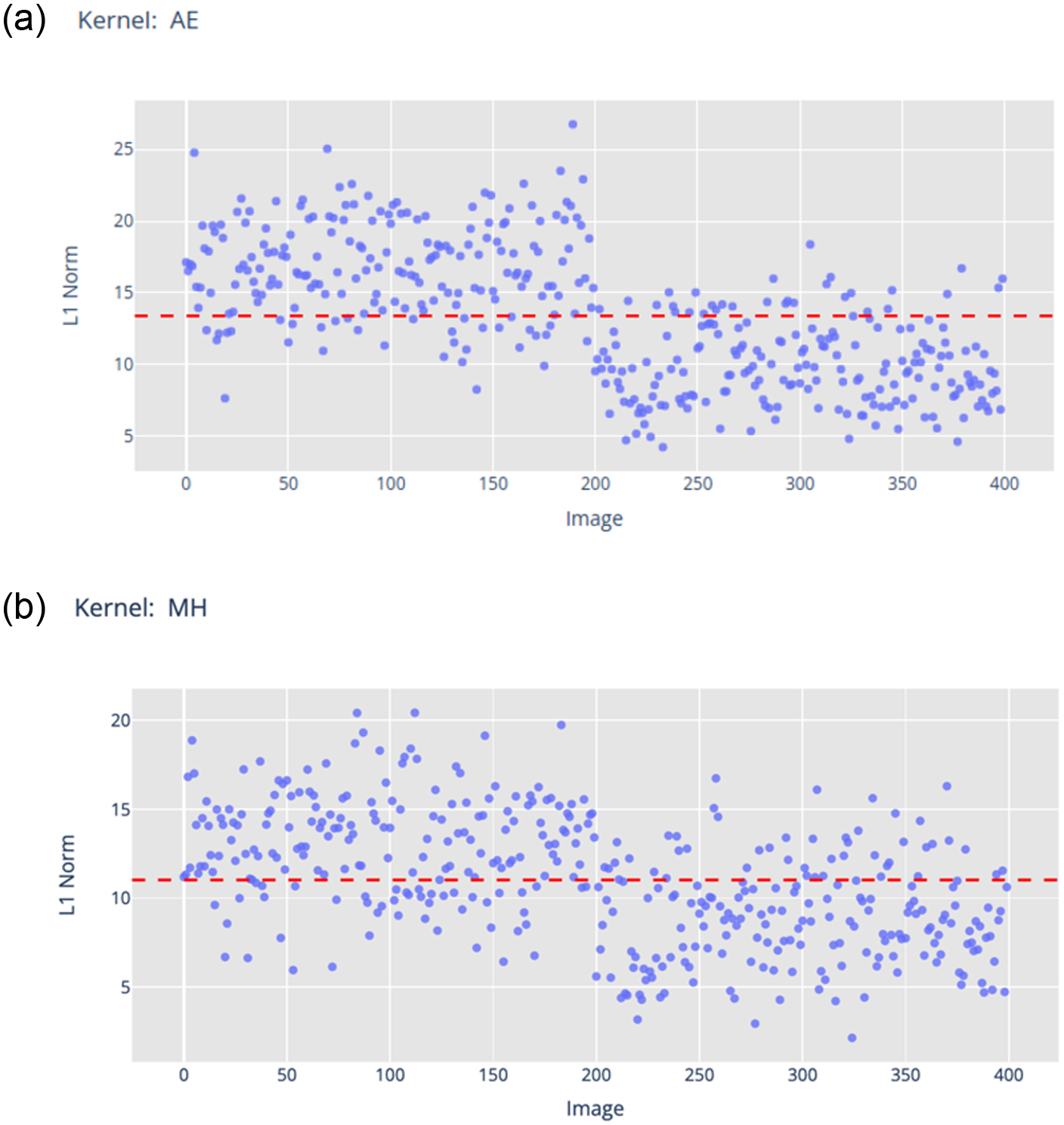

Supplement: S5 Fig — The first 200 data points are labelled as healthy and the next 200 as pleural effusion in the ground truth. A threshold value (red line) separates positive literals (above the line) and negative literals. (tif) [file pone.0293967.s007.tif]

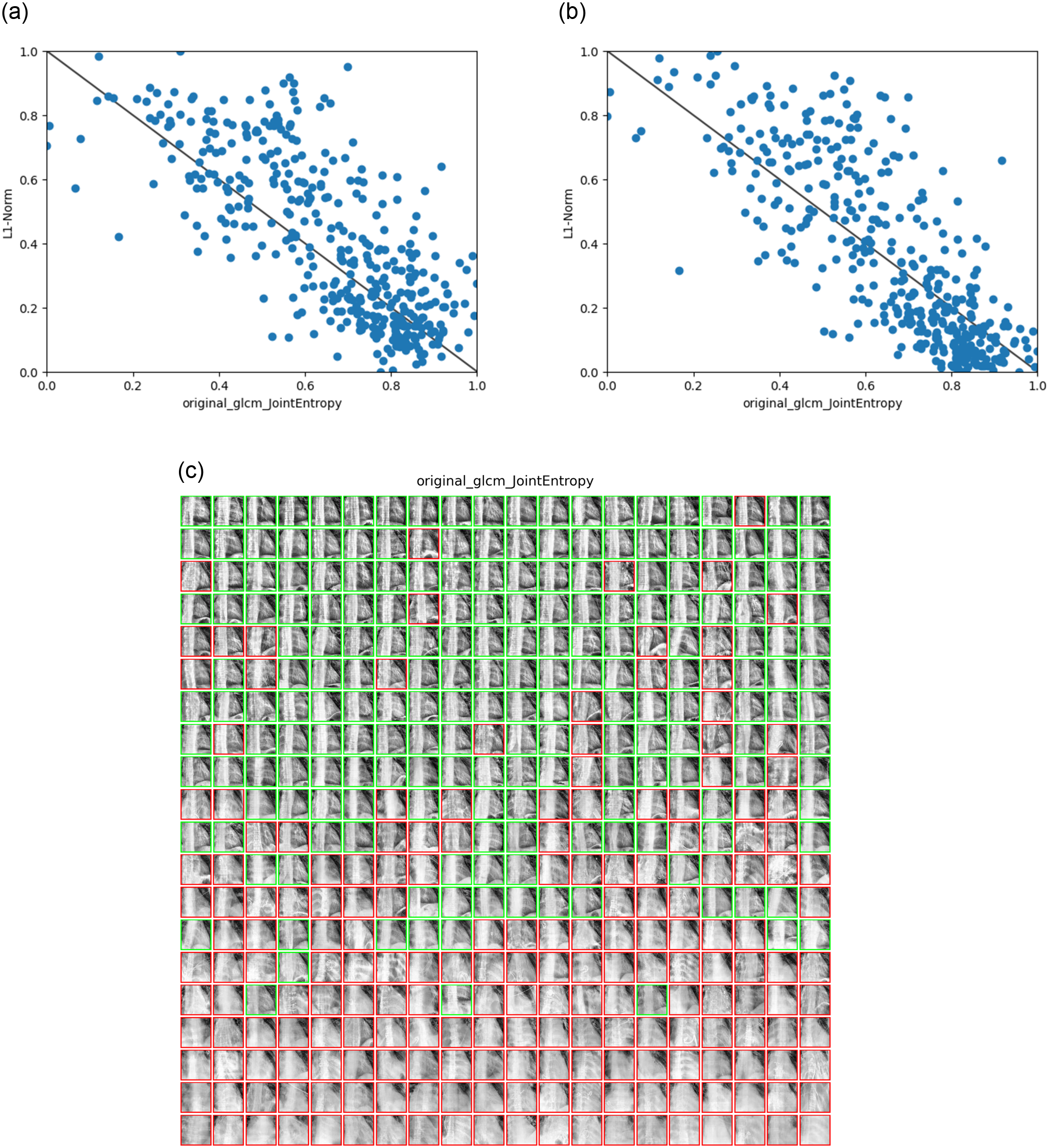

Supplement: S6 Fig — The first 200 data points are labelled as healthy and the next 200 as pleural effusion in the ground truth. A threshold value (red line) separates positive literals (above the line) and negative literals. (tif) [file pone.0293967.s008.tif]

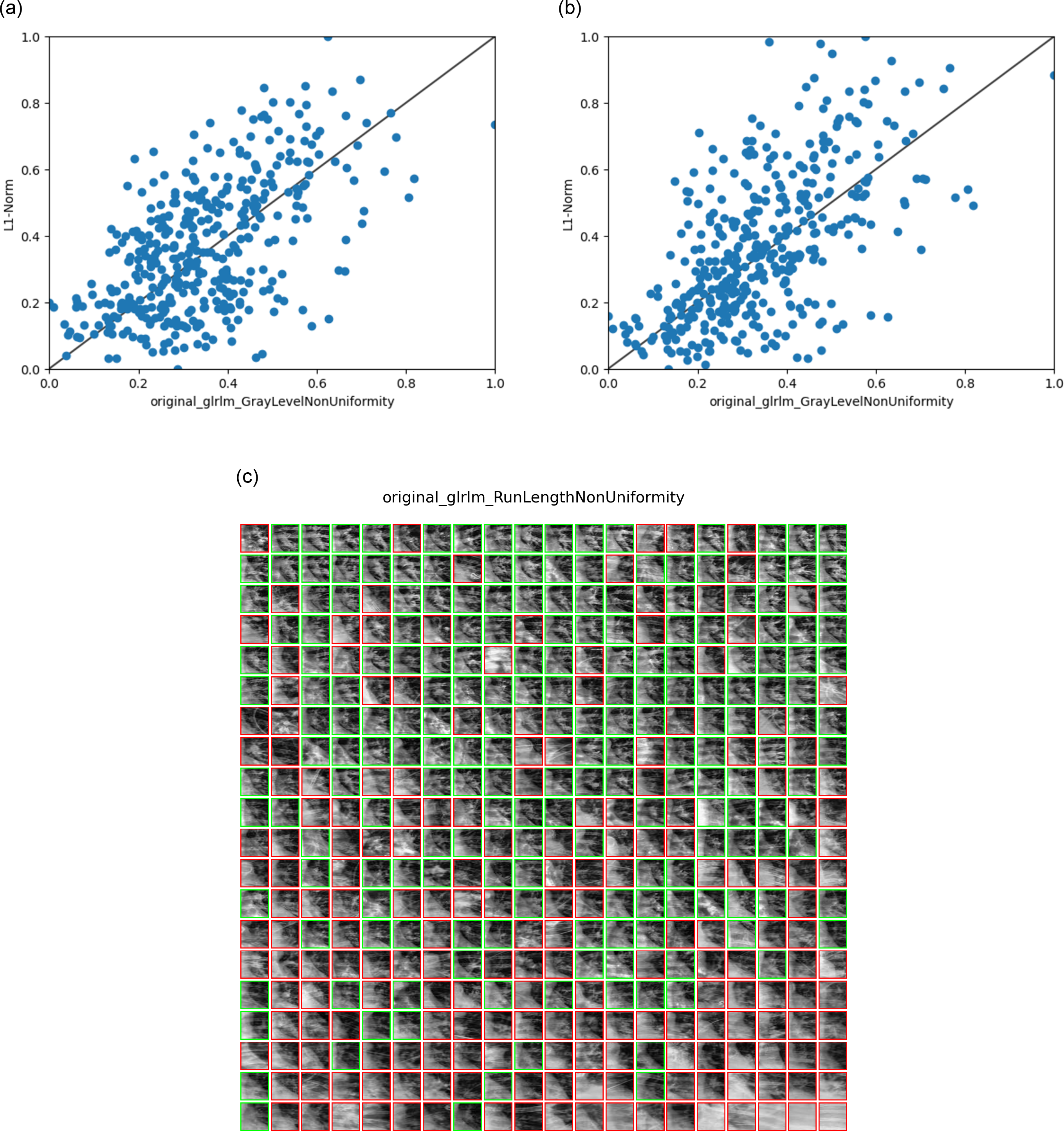

Supplement: S7 Fig — Correlation between Run Length Gray Level Non-Uniformity (GLRLM) with L1-Norms for (a) Kernel QT and (b) Kernel DM. Sub-figure (c) shows images of the Left Hilar region sorted row-wise from highest Run Length Gray Level Non-Uniformity (GLRLM) (top left) to lowest Run Length Gray Level Non-Uniformity (GLRLM) (bottom right). Those images with healthy as ground truth are outlined green while those with pleural effusion are outlined red. (tif) [file pone.0293967.s009.tif]

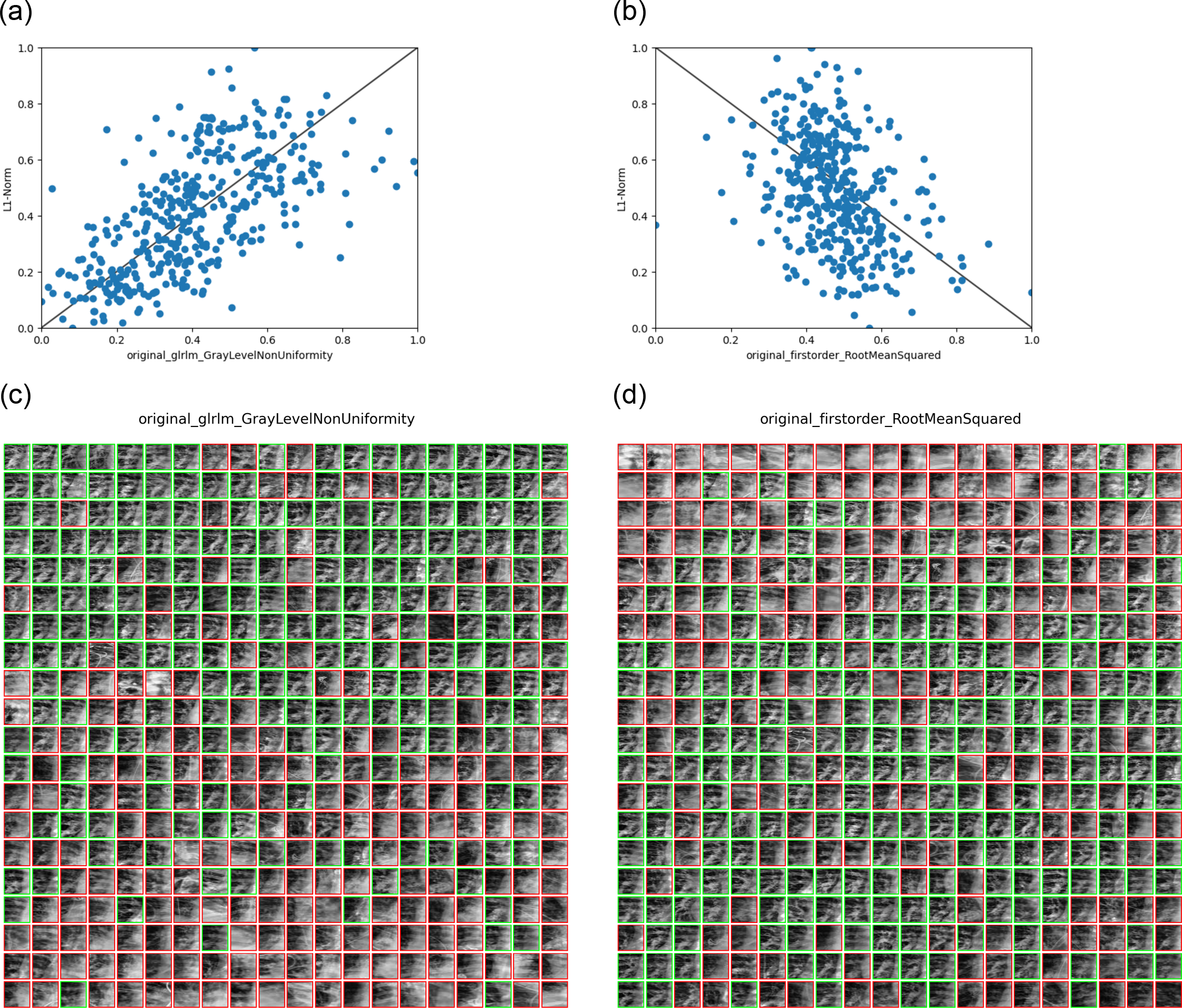

Supplement: S8 Fig — Correlation between (a) between Run Length Gray Level Non-Uniformity (GLRLM) and L1-Norms for Kernel AE, and (b) between First Order Pixel Intensity Root Mean Squared and L1-Norms for Kernel MH. Sub-figure (c & d) shows images of the Right Hilar region sorted by Run Length Gray Level Non-Uniformity (GLRLM) and Pixel Intensity Root Mean Squared row-wise respectively from highest value (top left) to lowest value (bottom right). Those images with healthy as ground truth are outlined green while those with pleural effusion are outlined red. (tif) [file pone.0293967.s010.tif]

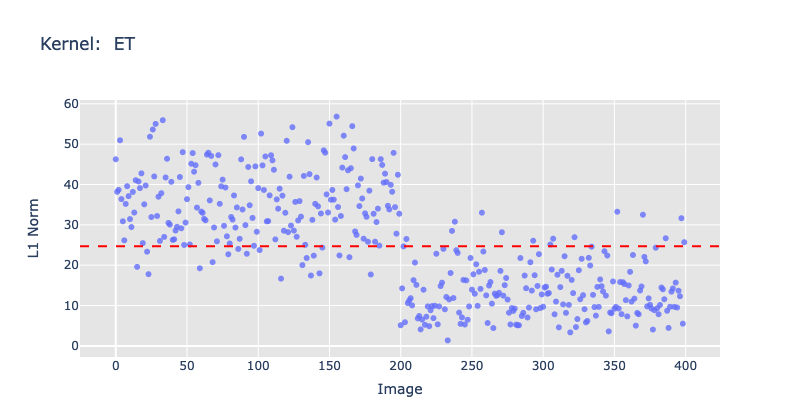

Supplement: S9 Fig — The first 200 data points are labeled as healthy and the next 200 as pleural effusion in the ground truth. A threshold value (red line) separates positive literals (above the line) and negative literals. (tif) [file pone.0293967.s011.tif]

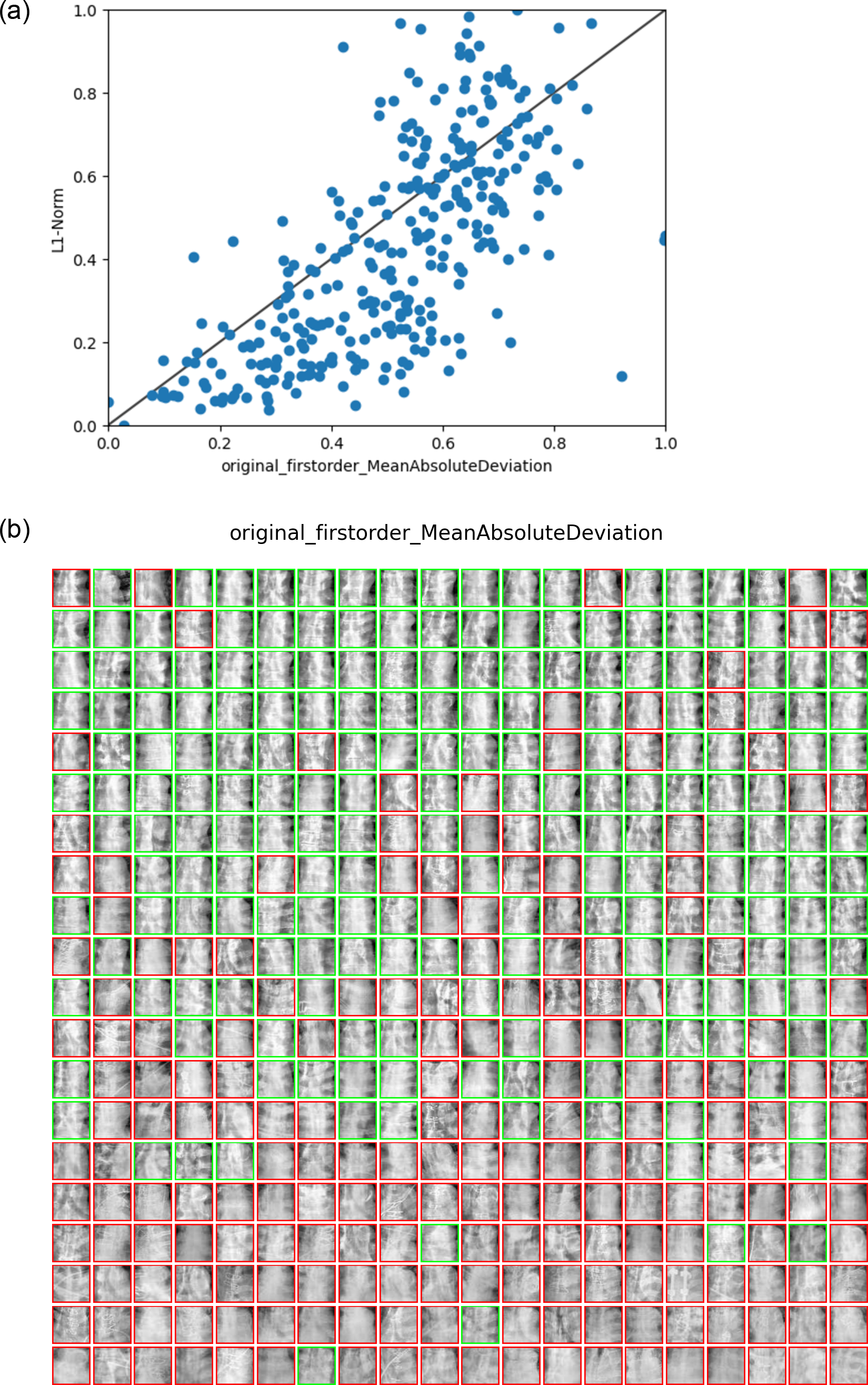

Supplement: S10 Fig — (a) A postive correlation between between First Order Mean Absolute Deviation (FOMAD) and L1-Norms for Kernel ET. Sub-figure (b) shows images of the Upper Mediastinum region sorted by First Order Mean Absolute Deviation (FOMAD) from highest value (top left) to lowest value (bottom right). Those images with healthy as ground truth are outlined green while those with pleural effusion are outlined red. (tif) [file pone.0293967.s012.tif]
